# Understanding the reminiscence bump: A systematic review

**DOI:** 10.1371/journal.pone.0208595

**Published:** 2018-12-11

**Authors:** Khadeeja Munawar, Sara K. Kuhn, Shamsul Haque

**Affiliations:** 1 Department of Psychology, Jeffrey Cheah School of Medicine and Health Sciences, Monash University Malaysia, Jalan Lagoon Selatan, Bandar Sunway, Subang Jaya, Selangor, Malaysia; 2 Department of Psychology, University of Wah, Wah Cantt, Pakistan; 3 Department of Teaching and Learning, College of Education and Human Development, University of North Dakota, Grand Forks, North Dakota, United States of America; University of Limerick, IRELAND

## Abstract

One of the most consistently observed phenomena in autobiographical memory research is the reminiscence bump: a tendency for middle-aged and elderly people to access more personal memories from approximately 10–30 years of age. This systematic review (PROSPERO 2017:CRD42017076695) aimed to synthesize peer-reviewed literature pertaining to the reminiscence bump. The researchers conducted searches in nine databases for studies published between the date of inception of each database and the year 2017. Keywords used included: reminiscence, bump, peak, surge, blip, reminiscence effect, and reminiscence component. Sixty-eight quantitative studies, out of 523, met the inclusion criteria. The researchers implemented a thematic analytic technique for data extraction. Four main themes were generated: methods of memory activation/instruction for life scripts, types of memory/life scripts recalled, location of the reminiscence bump, and theoretical accounts for the bump. The two prevailing methods of memory activation implemented were the cuing method and important memories method. Three types of memories/life scripts were recalled: personal/autobiographical memory, memories for public events, and life script events. The findings illustrate differing temporal periods for the bump: approximately 10–30 years for memories for important events, approximately 5–30 years for memories that were induced by word cues, and 6–39 years for studies using life scripts. In explaining the bump, the narrative/identity account and cultural life script account received the most support.

## Introduction

When examining the life span distribution of autobiographical memories (AMs), three phenomena are revealed. The first is childhood amnesia, or the limited recollection of AMs from a very young age, which is present in the life span retrieval curve as a steadily rising function between 0–8 years of age [1]. The second, the recency effect, dictates that memories recalled by most individuals are of recent events, and the frequency of these memories decline gradually [2]. Lastly, the reminiscence bump—also known as “the bump”—enhances memory recall from approximately 10–30 years of age by people over the age of 30 [3], and is considered one of the most robust findings in AM research [4]. As the reminiscence bump features deviation from the standard forgetting curve and forgetting functions [2, 3], it is recognized as a peculiar phenomenon and defining feature of AM [2, 3]. The reminiscence bump concept is included in most introductory psychology textbooks due to its significance to AM and the field of cognitive psychology [5–7].

The distribution of AMs across the adult life span is often studied through two major types of cueing techniques: the word cuing method and the important memories method [8]. There are various other methods of activating memories, some of them are; writing home diaries, free-recall of public and private items of news, and answering factual, semantic, general-knowledge, multiple-choice questions about the Academy Awards, the World Series, and current events [9–11]. The word cuing method, originally developed by Francis Galton, was later modified by Crovitz and Schiffman [12, 13]. In this technique, participants retrieve and report memories in response to word cues commonly used in everyday conversation [14–17]. The word cuing method was used rigorously in investigating personal memories during the 1970s and 1980s [12, 18–20]. In the important memories method, participants are instructed to retrieve and report the most important memories from their life [8, 15, 21, 22], especially the vivid ones [23–25].

A significant amount of research emerging in the last two decades, claims that the previously found reminiscence bump in AM also extends to public events [26]. Research shows that public events which occurred during adolescence or early adulthood, approximately from the age of 12 to 29 years, are preferentially recalled [27]. This phenomenon is assessed through two major methodologies: the first asks participants to name significant events from recent history [28], and the second assesses participants’ level of factual knowledge of specific events [11].

Researchers propose various theoretical accounts to explain the reminiscence bump [8], including the: cognitive account, cognitive abilities account, cultural life script account, and narrative/identity account. The cognitive account postulates that it is simply the novelty of many events occurring in the second and third decades of life that is the major factor leading to enhanced memory recall from this period [29]. According to the cognitive abilities account, people become better equipped to learn, process, and retain information as they move into adolescence and early adulthood due to the maturation of the brain, which leads to maximal cognitive and neurological functioning [30]. According to the cultural life script account, individuals recall more events from the second and third decades of life because of cultural prescriptions and expectations present in the life script [31–33].

The narrative/identity account states that events occurring during adolescence and early adulthood are vital to the development of the individual’s adult identity. It is this time when an individual engages in activities and relationships that define who the person will finally become, and how they narrate the stories of their lives [11, 24, 34, 35]. By the time an individual reaches this period of life, the effect of novel experiences on long-term memory, recognition, self-identity, and the development and consolidation of goals, have typically been demonstrated [36, 37]. Experiences acquired during this period are integrated into an individual’s lifelong narratives, thus they are more easily recalled later in life. The critical role of early adulthood AMs in identity formation is illustrated in neuropsychological and developmental research [38, 39]. The working self is viewed as playing a major role in organizing AMs; and events during this critical period are used as identity markers for the remainder of life each time AMs are reconstructed [40]. Broadly, the self (or identity) is conceptualized as a multidimensional and complicated set of self-related processes and schema [36, 41, 42].

The research identifies two components of the reminiscence bump: one relating to social identity (i.e. AMs corresponding chiefly to public events individuals experienced when ages 10 to 19 years old), and the other relating to personal identity (i.e. AMs corresponding to personal events that happened between the ages of 20–29) [10]. While social identity develops, individuals associate themselves with specific cultural, social, political, and/or religious groups with whom they have similar goals and desires [43]. Alternatively, during the development of personal identity, desires and goals towards establishing interaction with significant others and forming intimate relationships are developed [36]. The enhanced recollection of social events results in the formation of a reminiscence bump for the ages of 10–19 years, while developing close personal relationships results in a bump for the ages of 20–29 years [10].

Cultural life scripts are stereotypical episodes comprising multiple events in a specific order, with every event allowing succeeding events to occur [44]. They are a series of events which occur in a particular sequence and characterize a prototypical life span in a certain culture [31–33]. The scripts have slots with specific conditions for what is allowed to fill them [45]. The slots in cultural life scripts are culturally significant transitional events likely to occur in a prototypical life span in a certain culture [46]. According to one study, an important life script characteristic is that it represents a culturally shared part of our semantic knowledge; not the outcome of a few personal experiences [32]. Another research study opposes this finding, reporting that cultural life scripts are not part of our shared semantic knowledge [47].

The cultural life script account is based on observation; as reported by Neugarten, Moore and Lowe (1965): Certain age norms are present in every society which organize the expectations, and structure the behaviour, of individuals [46]. There are prescriptive timetables in every culture for the arrangement of significant life events (e.g., finish school, get a job, get married, and have the first child) and the individuals of that particular culture are aware of these age norms [48]. Individuals also manage their own timing for the events on these timetables, and assess if they are achieving significant events earlier, or later, than anticipated [49]. The mechanisms underlying the bump for public events may be different than the mechanisms underlying the bump in autobiographical memory.

The earlier review papers summarized the studies on retention function, reanalyzed the previous findings, reviewed the temporal location of the bump according to different cueing methods, and assessed the current theoretical accounts of the bump in light of the temporal locations of the bump [2, 4, 8, 26]. These studies presented different bump periods for different methods of memory activation. The past reviews summarized the empirical evidence on the bump from non-clinical sample and excluded findings from the clinical samples as well as immigrants as they were interest in "the location of the bump in the general population" (p.67, Koppel & Berntsen, 2015). The differences in location of the bump with respect to cuing methods has already been shown in the past research studies [15, 16]. However, the mechanisms underlying the bump for autobiographical memory activated by different cuing methods (e.g., word cuing method and important memory method) might be different as well as the mechanisms underlying the bump for different types of responses (autobiographical memory vs. Life script events) could also be different.

As the bump is one of the most robust findings in autobiographical memory research, the present review paper added to the empirical body of literature on the bump and attempted to take a step a little further by including and reporting past studies’ findings on: general population, clinical samples, immigrants, or any other samples. Furthermore, no restriction was applied on methods of memory activation and research studies assessing the reminiscence bump through methods other than word cuing methods and important methods were also screened for eligibility. The last review paper on bump was published in 2015, therefore, this review was conducted with the belief that more recent and latest findings from the studies could be assessed and summarized.

The authors attempted to present a thorough summary of all the existing primary research studies on the bump, tried to establish the state of existing knowledge and reviewed the bump for various types of memories apart from autobiographical memories, for instance, flashbulb memories and memories public/private events. The authors developed a clearly defined, predetermined eligibility and relevance criteria for including research studies; the methodology was reproducible, transparent and systematic; carried out a meticulous search to identify all suitable studies; assessed quality of included studies, and systematically synthesized all the evidence in the form of figures and tables. The authors tried to limit selection bias and random error which have been found to mislead the reviews [50, 51], and attempted to present a reliable summary of the existing knowledge.

In the existing literature, there is currently no systematic review on the reminiscence bump which implements the guidelines proposed in the Preferred Reporting Items for Systematic Reviews and Meta-Analyses (PRISMA) statement [52]. However, a general review of the location of the reminiscence bump across different methods of memory activation provides additional references and descriptions [8]. Although there are several theoretical accounts for the bump for various kinds of memories, and all receive some support in the literature, it is necessary to investigate the relative plausibility of each account. The temporal location of the bump is not presented consistently in studies using varied methods of memory activation. The mechanisms underlying the bump may be different across different memory domains and types of memory assessments.

Discovering the most likely temporal location of the reminiscence bump is one of the authors’ aims in conducting the systematic literature review presented in this paper. Another goal of the researchers is to ascertain which theoretical account for the reminiscence bump received the most overarching support, by examining the literature for reported temporal locations of the bump in relation to the methods used to activate different types of memories in participants from various countries.

## Method

The authors implemented the PRISMA statement guidelines in designing this systematic review [52]. After the researchers developed the review protocol, they registered the protocol in PROSPERO (International prospective register of systematic reviews; please see S1 File) prior to the commencement of the review (registration number CRD42017076695) [53].

### Eligibility criteria

Eligible articles were required to present original research on the bump from qualitative, experimental, quasi-experimental, non-experimental, observational, or mixed-method studies. Neither language of published article, nor sample age group was limited. Only articles published in peer-reviewed or refereed journals were selected. Grey literature and articles that did not mention the reminiscence bump or its synonyms in their titles or abstracts, were excluded. No restriction was imposed on date of publication to allow for a comprehensive background on—and theoretical progression for—the reminiscence bump over time, as presented in the research.

### Systematic search strategy

The researchers conducted a systematic search to locate primary source articles. Synonymic keywords searched in each database, using the Boolean OR operator [54] and wildcard features (e.g. placing an asterisk at the end of a root word to account for a variety of word endings), included: reminiscence*, bump, peak, surge, blip, reminiscence effect*, and reminiscence component (please see S1 Table). The search strategy combined these synonymic keywords (with OR) to search the following 9 databases for articles published, or added, to the databases between the date of inception of each database and 2017: Ovid MEDLINE, Ovid Embase, Ovid Emcare, CINAHL Plus (EBSCOhost), Proquest Central, PsycInfo, Scopus, Pubmed, and ScienceDirect (please see S2 Table).

**Fig 1:**
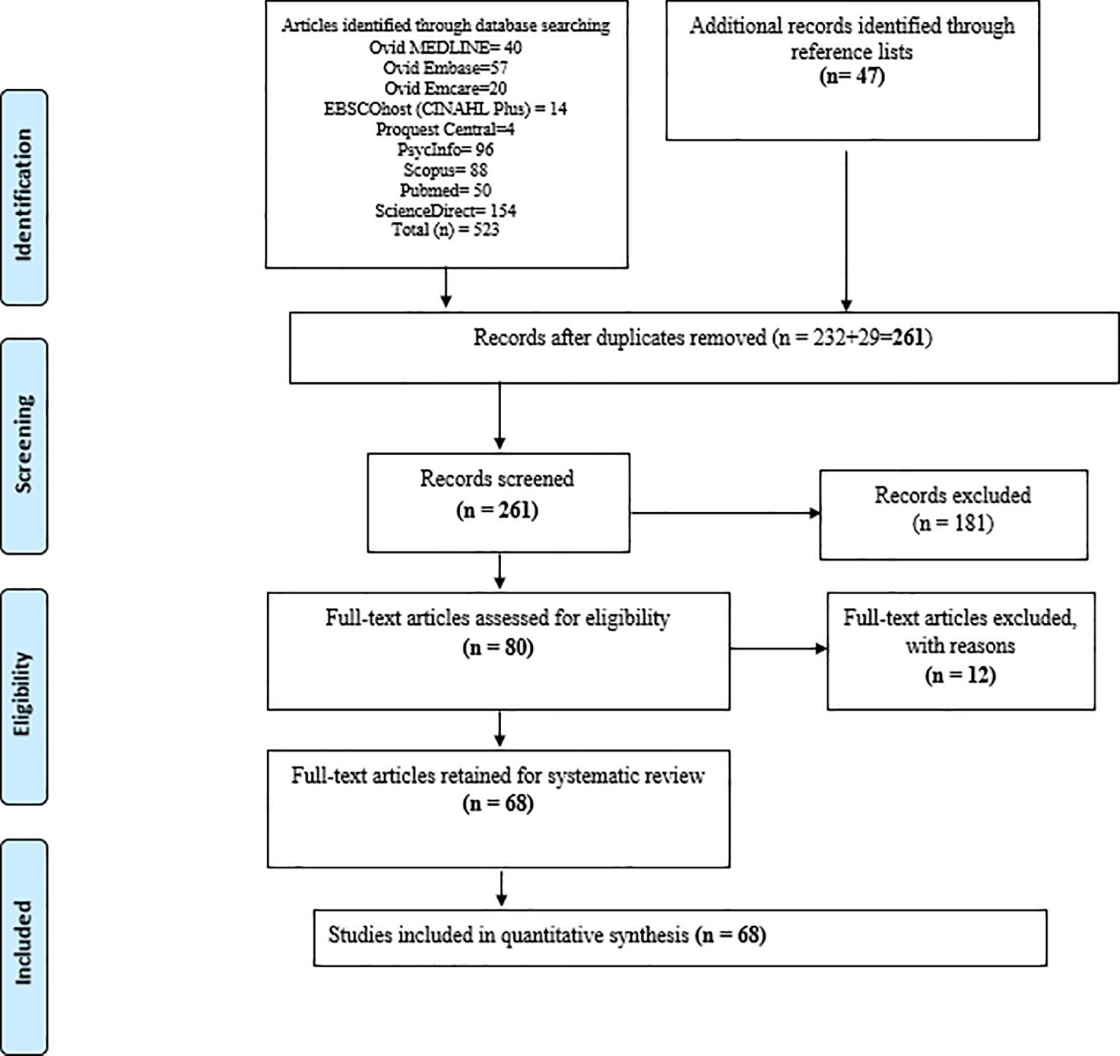
PRISMA flow diagram showing process of study selection for inclusion in systematic review.

The researchers retrieved a total of 523 records through this search strategy. They then “hand searched” the 523 articles’ reference lists to obtain further relevant studies, yielding 47 additional citations (Fig 1). After the researchers removed duplicates, 261 articles remained.

The 261 article records were imported into the EndNote reference/citation manager and screened for eligibility/inclusion in the review. The researchers screened titles and abstracts for inclusion criteria, removing 181 studies that did not meet the criteria and retaining 80 full-text articles to assess for eligibility. After assessing the 80 full-text articles, 12 articles were excluded for not meeting inclusion criteria (e.g. not published in peer reviewed journals, or format was a brief report and not a research article). The researchers hand searched the remaining 68 articles’ references for relevant articles. No additional articles were included from this search. The final group of 68 studies was assessed for methodological quality, after which the researchers performed data extraction and synthesis.

### Methodological assessment

The 68 quantitative studies were assessed for quality using the 14 criteria developed by Kmet, Lee, and Cook [55]. No qualitative or mixed-method studies were present. An overall rating (from 0 to 1) was assigned to every study; higher numerical ratings indicated higher quality. Previous systematic reviews employing the QualSyst quality assessment protocol required a minimum threshold score of 0.55 for study inclusion [56]. Other reviews included studies falling within the range of 0.74 to 0.91[57]. The lowest quality rating of studies included in this review was determined to be 0.54, therefore all studies were considered eligible for inclusion. To minimize the risk of bias, two reviewers worked independently to screen studies and extract and synthesize data. Disagreements were settled by applying the 14 criteria [55] (please see S3 Table).

### Data collection and extraction

The researchers used forms to extracted the data for retrieving relevant information to assess the aims of the review [58]. They placed extracted information under appropriate sections corresponding to: author, year, country; study objective; sample size (N), and findings (Table 1)

**Table 1:** Summary of studies about the reminiscence bump ^1^ (N = 68).

**Serial. No.**	First Author/Year/ Country ^2^	Objective	Sample size (n)	Findings
**1.**	Alea, 2014 Trinidad and Tobago [59]	Examining the life-script account for the reminiscence bump	N = 100; range: 31–59 years	Two reminiscence bumps were found for both positive and negative events: (a) 6–15 years old, and (b) in the mid-twenties. The first bump comprised mostly ordinary events, regardless of participant age. The second contained mostly unusual events, regardless of valence; people ≥40 years exhibited a bump for negative events.
**2.**	Berntsen, 2004 Denmark [32]	Replicating earlier findings on age norms for emotional events in a large stratified sample Providing direct evidence for the existence of life scripts for a series of events	Study 1: 1485 respondents (age range 20–99 years) Study 2: 103 undergrads (87 women, 16 men; mean age, 26.4 years; range: 21–51 years)	Showed an increase in transitional events between 15–30 years old that were associated with narrower age ranges and more positive emotion than events outside this period. Only positive events increased between the ages of 15–30. Evidence of a shared life script for transitional events biased to favor positive events and events expected to occur in the period of the bump. Most positive events were estimated to occur between 15–30 years old; the distribution of negative events was relatively flat.
**3.**	Bohn, 2011 Denmark [60]	Examining children’s representations of possible events in their personal futures	Study 1: 162 middle-class children (mean ages for third graders 10.01, SD = 0.49; mean ages for eighth graders 14.62 years, SD = 0.30; 51% men, 49% women) Study 2: 20 eighth graders (11 men, 9 women; mean age = 14.77 years, SD = 0.40)	Events in these life stories were mostly life-script events, and their distribution showed a clear bump in young adulthood. The events generated consisted mostly of non-life-script events, and those events did not show a bump in young adulthood.
**4.**	Cappeliez, 2008 Canada [9]	Examining older adults’ dreams with content dated during the bump period, first in terms of central concern, and second in terms of type of reminiscence	30 older women (mean age = 65; range = 60–77)	Dreams were found to be characterized by content related to personal goals and akin to the integrative type of reminiscence, supporting the theory that personal memories of the bump period remain salient—even in the dreams of older adults, because they relate to the development of goals of the self and identity—and that older adults use personal data to (re)construct a coherent and meaningful self.
**5.**	Chu, 2000 UK [61]	Examining differences in the age distributions of odor-cued and verbal label-cued AMs among older participants	Odor condition: 22 subjects (11 men, 11 women; mean age = 69.4) Label condition: 11 subjects (4 men, 7 women; mean age = 69.6)	The bump for label cues was found to peak between ages 11–25. The odor-cued memory distribution peaked at 6–10 years and decreased linearly thereafter. In the earliest age interval, 6–10 years, the proportion of AMs retrieved in response to odor cues was significantly greater than that for label cues.
**6.**	Conway, 1999 Bangladesh [62]	Identifying the timing of the reminiscence bump in a sample of Bangladeshi people	106 participants (80 men and 26 women, mean age = 47)	A reminiscence bump was found for the period 10–30 years. It was prominent in the younger group, but less so in the older group, who also showed a bump from age 35–55. This latter bump corresponded to a period of national conflict between Pakistan and the Bengali people, resulting in the formation of an independent Bangladesh.
**7.**	Conway, 2005 Japan, Bangladesh, England, China, USA [63]	Exploring memory and self-cross-cultural differences, in particular, examining an aspect of AM that is often observed and known to be closely associated with self: the reminiscence bump	208 participants: 33 from Japan, 40 from Bangladesh, 27 from England, 54 from China, 54 from US. Age range: 38–60 (overall mean age = 52 years)	Periods of childhood amnesia and the reminiscence bump were the same across cultures. Memories from the Chinese group consisted of interdependent self-focus (i.e., were of events with a group or social orientation). Memories from the U.S. group showed an independent self-focus (i.e., were of events oriented to the individual).
**8.**	Copeland, 2009 USA [64]	Examining the forgetting curves for information read in a novel	38 participants: 24 undergraduate and graduate students, 14 undergraduates	A clear effect of primacy along with two reminiscence bumps: one observed around the age of 20, typical in studies of the bump; and one bump later in life at the time of an important life transition.
**9.**	Davison, 2008 UK [65]	Applying an AM framework to the study of regret	Study 1: 60 participants (ages 60–69; mean age = 65; SD = 3.1) Study 2: 71 participants (45 women; 26 men). Two age groups recruited: • 40s (n = 41); mean age = 44.4; SD = 3.4 • 60s (n = 30); mean age = 64; SD = 2.9	There was reminiscence bump for general, but not for specific, regrets. Recent regrets were more likely to be specific than general in nature.
**10.**	Demiray, 2009 Turkey [66]	Replicating the reminiscence bump using a life history timeline method Extending reminiscence bump research to a Turkish sample Empirically examining the recently proposed life story account for the reminiscence bump.	72 participants (40 women and 32 men, ages 52–66; mean age = 58.25; SD = 3.86)	A bump was found between the ages of 10–30 using a life history timeline method. A life story account of the bump is supported by showing that bump memories are perceived as more novel, were more likely to be distinctive events, were rated as more important for identity development, and were more likely to be transitional events than memories from other parts of the lifespan distribution.
**11.**	Denver, 2010 USA [67]	Examining flashbulb memories acquired from different points in the lifespan in younger and older adults	Study 1: 67 participants: 39 younger adults, ages 20–42 years, mean age = 23.3, and SD = 3.7; and 26 community-dwelling older adults, ages 59–89, mean age = 73.9, and SD = 7.0 Study 2: 58 participants: 39 younger adults, mean age = 23.3, and SD = 3.7; and 19 older adults, mean age = 73.7, and SD = 7.6	Older adults’ flashbulb memories created during the reminiscence bump period were very vivid and highly accessible, and showed a clear reminiscence bump between the ages of 10–30 years
**12.**	Dickson, 2011 USA [68]	Examining if a reminiscence bump is evident when memory cues prompt recall of surprising and unexpected events	Study 1: College students, ages 17–33 (n = 198). Mean age = 18.67; SD = 1.76 Study 2: Older adults, ages 60–93 (n = 259). Mean age = 70.65; SD = 7.77 Study 3: College students, ages 17–26 (n = 196). Mean age = 18.59; SD = 0.99 Study 4: Older adults, ages 60–89 (n = 227). Mean age = 68.65; SD = 6.81	Older adults recalled: positive and negative, surprising positive and surprising negative, or highly expected and highly unexpected events. Adults’ memory distributions were compared with distributions of predicted life events generated by undergraduates. Reminiscence bumps were found not only for memories of positive and expected events, but also for memories of surprising and unexpected events.
**13.**	Ece, 2014 Turkey [69]	Exploring the impact of suppressing typical life events on the reminiscence bump in life script and AM distributions	142 participants, ages 45–65. (mean age = 51.82; SD = 4.80	The reminiscence bump disappeared in AM distribution. In life script distribution, it disappeared from 21–30 years, whereas it was reduced between 16–20 and 31–35 years.
**14.**	Elnick, 1999 USA [70]	Advancing the understanding of how people represent who they are through AMs	220 participants (104 men, 116 women) ages 40–87 (mean age = 59.1; SD = 12.2)	Memories demonstrated the bump in early adulthood. The central domains represented in this era involved events with family and relationships followed by those related to education and work.
**15.**	Erdoğan, 2008 Turkey [71]	Testing the generality of the life script by looking at the effects of culture, gender, and cohorts	200 participants (114 women, 86 men, ages 18–34 (mean age = 20.08; SD = 2.07)	A clear life script was obtained containing more positive than negative events; there was a stronger agreement about the timing of positive, than of negative, events. Many aspects of the life script, but not the bump, changed depending on the age of the individual for whom the script was constructed (newborn vs. elderly).
**16.**	Fitzgerald, 1988 USA [24]	Developing an explanation for the phenomenon of the reminiscence bump	Study 1: 31 participants (15 men, 16 women) ages 60–75 (mean age = 67.2) Study 2: 51 participants (25 men, 26 women) ages 62–75 (mean age = 68.7)	Findings revealed that reminiscence effects reflect the availability of a pool of vivid memories from a given era.
**17.**	Fitzgerald, 1996 USA [35]	Ascertaining the distribution of life story memories for both younger and older adults	Group 1: 45 participants, 23 men and 22 women, ages 30–46 (median age = 36) Group 2: 45 participants, 20 men and 25 women, ages 60–75 (median age = 66)	Adults reported a large proportion of memories from adolescent and young adult periods. Younger and older adults showed similar patterns of sampling from that period.
**18.**	Fromholt, 1991 Denmark [72]	Analyzing changes in the way memories are recalled by patients with dementia to identify features of AM function that are especially vulnerable to degenerative brain processes	60 participants total, ages 71–89. Experimental group: 30 volunteers with dementia (5 men and 25 women; mean age = 80.5). Control group: 30 volunteers (12 men and 18 women; mean age = 78.3	The chronological distribution of memories across the life span in both groups showed a peak in adolescence and early adulthood, a decrease in mid-life, and an increase in recent years. This distribution is similar to the chronological pattern reported for vivid memories. The distribution in the demented group was more flat.
**19.**	Fromholt, 2003 USA [73]	Providing information on the influence of age, dementia, and depression on AM in late life	Experiment 1: 15 participants (11 women) Experiment 2: 22 participants (17 women); mean age = 100 years, seven months (range = 100 years, 0 months to 101 years, nine months)	The life-narrative method produced relatively more bump memories. The life-narrative distributions were similar to those obtained from 80-year-old adults without clinical symptoms and from 80-year-old Alzheimer's dementia and depression patients. The centenarians had an additional 20-year period of relatively low recall between the bump, recency components, more emotionally neutral memories and fewer and less detailed memories.
**20.**	Gidron, 2007 Netherlands [74]	Exploring the relationship between overgeneral memory biases in the context of the distribution of AMs found in later age, and depression	25 participants (12 men, 13 women), ranging in age from 65–89 (mean age = 77.92; SD = 6.5 years)	The reminiscence bump was found to be significantly and inversely correlated with depression.
**21.**	Glück, 2007 Austria [22]	Addressing the theoretical issue of causes for the reminiscence bump Examining the adequacy/inadequacy of the life story account	659 participants, ages 50–90 years. Three age groups: ages 50–59 (n = 285); ages 60–69 (n = 195); and ages 70–90 (n = 171)	Only high perceived-control positive events showed a bump, and were rated as more influential on later development than were events showing any other combination of valence and perceived control.
**22.**	Haque, 2010 Malaysia [75]	Explaining the reminiscence bump for emotionally charged AMs among Malaysian participants	Study 1: 189 middle-aged older participants. Women = 111 (ages 50–76; mean age = 56.0 years; SD = 6.0). Men = 78 (ages 50–90; mean age = 58.0, SD = 8.0) Study 2: 92 undergraduate students. Women = 73, Men = 19. Ages 17–23 (mean age of women = 19.74, SD = 1.21; mean age of men = 19.53, SD = 0.77)	The findings revealed bumps in both life script and retrieval curves for the memories deemed to be the happiest, most important, most in love, and most jealous. A reminiscence bump was also noted for success, although it occurred later in the lifespan than other bumps.
**23.**	Holmes, 1999 England [10]	Examining differences between age groups in both type and content of knowledge recalled from the period of ages 10–19 years old	Experiment 1: 100 participants, ages 30–70, separated into four groups of 25 by age: 30–39 (mean age = 35), 40–49 (mean age = 45) 50–59 (mean age = 55), and 60–70 (mean age = 64) Experiment 2: 100 participants, ages 30–70 (mean age = 50) in four groups of 25 by age: 30–39 (mean age = 36), 40–49 (mean age = 45), 50–59 (mean age = 54), and 60–70 (mean age = 64)	The peak recall for public news items was found in the period from ages 10–19, whereas peak recall of private news items occurred in the period from ages 20–29. These two components of the reminiscence bump reflect, respectively, a period of formation of generation identity in the second decade of life and a period of formation of intimate relationships in the third decade.
**24.**	Jansari, 1996 UK [76]	Exploring the reminiscence bump: the disproportionately higher recall of early-life memories by older adults	Experiment 1: 82 participants (63 women; 19 men). Three age groups: 36–40, 46–50, & 56–60 (mean ages = 18.2, 49.2, & 66.8, respectively) Experiment 2: 24 new participants 45–60 years of age (mean age = 52.3 years)	Findings of experiment 1 revealed the appearance of a bump in younger participants. In experiment 2, memories from early life were more easily retrieved, but it was not due to differences in subjective qualities. A higher proportion of memories for first-time events were identified from early life, and they were more easily retrieved.
**25.**	Janssen, 2003 Netherlands [77]	Researching the distribution of AM through the Galton-Crovitz cueing method	1,587 participants (827 men; 760 women), ages 10–70 (mean age = 39.89). Six age groups.	Findings revealed a bump from 13–18 years old, which is an estimate for the age interval during which the expected number of encoded memory representations reaches its highest value.
**26.**	Janssen, 2005 Netherlands and USA [78]	Investigating the age distribution of AM using the Galton-Crovitz method through the Internet	2,000 participants, ages 11–70. Mean age = 38.38 (SD = 13.74)	Strong evidence was found for a bump with peaks at ages 15–18 for men, and 13–14 for women. Americans showed a tendency to report older memories than Dutch participants. Age group and level of education did not influence lifetime encoding information.
**27.**	Janssen, 2007 Netherlands [79]	Examining if the bump is caused by differential encoding or re-sampling	Participants from the Netherlands (n = 1,279), U.S. (n = 406), U.K. (n = 104), Belgium (n = 66), Australia (n = 64), Canada (n = 54), and other countries (n = 188). Mean age = 35.57 (SD = 14.46)	Temporal distributions showed reminiscence bumps. The distribution of favorite records had the largest reminiscence bump. The results suggest that differential encoding initially causes the reminiscence bump and that re-sampling increases the bump further.
**28.**	Janssen, 2008 Netherlands [80]	Examining memory for public events to investigate why personal events are encoded better in adolescence and early adulthood than in other lifetime periods	1,334 participants, ages 16–75. Mean age = 42.9. Six age groups: 16–25 (n = 266), 26–35 (n = 236), 36–45 (n = 259), 46–55 (n = 338), 56–65 (n = 179), 66–75 (n = 56)	The participants answered questions correctly about events that occurred in the period in which they were between 10–25 years old. The bump was more pronounced for cued recall than for recognition.
**29.**	Janssen, 2008 Netherlands [30]	Exploring the causes of the reminiscence bump	3,492 participants, ages 16–75. Mean age = 42.3 (SD = 14.9)	The reminiscence bump consisted of relatively fewer novel, emotional, and important positive or negative events.
**30.**	Janssen, 2011 Japan [81]	Investigating the temporal distribution of word cued memories and how it is affected by the increased recall of recent events	50 participants, ages 16–65. Experiment: Polish participants (N = 1,089), more young adults (16–40 years, n = 961) than middle-aged adults (41–65 years, n = 128)	A model was proposed that: estimates a retention function based on the 10 most recent years from the observed distributions, and divides the observed distributions by predictions derived from the estimated retention function. It demonstrated that AM temporal distribution of participants younger than 40 contained the reminiscence bump.
**31.**	Janssen, 2011 Netherlands [82]	Examining whether AMs from adolescence and early adulthood are recollected more than memories from other lifetime periods	2,341 participants (739 men; 1602 women) ages 16–75 (mean age = 47.77; SD = 14.31)	Most memories came from the period of 6–20 years old, showing a bump. The memories from this period were not relived more often, or recalled more vividly, compared to recent events. Older adults reported a stronger recollective experience than younger adults.
**32.**	Janssen, 2012 Netherlands [83]	Examining the robustness of the reminiscence bump by looking at participants' judgments about the quality of football players	619 participants, ages 16–80. Mean age = 47.74 (SD = 15.31). Seven age cohorts by birth year: 1926–1935, n = 20; 1936–1945, n = 69; 1946–1955, n = 162; 1956–1965, n = 152; 1966–1975, n = 87; 1976–1985, n = 71; 1986–1995, n = 58	Participants frequently named football players who reached the midpoint of their career when the participants were adolescents (mode = 17). The results indicate that the reminiscence bump can also be identified outside the AM domain.
**33.**	Janssen, 2015 Australia [84]	Examining if people have culturally shared expectations about the timing of important public events	Condition 1: 107 participants, ages 16–28; 83.2% women (mean age = 18.87; SD = 2.01) Condition 2: 102 participants, ages 17–29; 73.5% women. (mean age = 20.96; SD = 3.00)	No support for cultural life scripts as an explanation for the bump in the memory for public events was found. Most public events were expected to occur before the reminiscence bump period.
**34.**	Ju, 2016 USA [85]	Examining the reminiscence bump in a new context: reactions to nostalgic advertising	168 participants sampled to represent two age cohorts: Gen X (n = 89); Boomers (n = 79). Gen X mean age = 33.28 (SD = 2.52), and late-stage boomers mean age = 53.47 (SD = 2.36)	Supports hypotheses that bump focused advertisements (ads) show higher diachronic relevance and elicit greater purchase intent than either non-bump past ads or present-focused ads. Greater purchase intent after viewing the bump-focused ad was shown to be mediated by the diachronic relevance of the ads.
**35.**	Kawasaki, 2011 Japan [86]	Examining the temporal distribution of AMs of Japanese young and middle-aged adults	252 participants, ages 16–65	A bump was identified in memories of young adults. The bump location of for young adults was 5–13 years of age, and for middle-aged adults, 6–15.
**36.**	Koppel, 2014 USA [87]	Testing the existence of normative youth bias	Study 1: 200 participants ages 18–81, mean age = 38.1, SD = 16.1 (63.0% men; 37.0% women) Study 2: 198 participants ages 18–71, mean age = 32.7, SD = 12.4 (60.6% men, 37.9% women)	A marked peak was found in young adulthood (i.e., ages 11–30), when the most important public event of a hypothetical person’s life would be expected to occur.
**37.**	Koppel, 2016 Denmark [88]	Comparing the bump for AMs versus the bump for memories of public events	42 participants, ages 40+ years. Mean age = 57.93 in the autobiographical event condition (range = 41–69; SD = 8.21) and 57.90 in the public event condition (range = 40–69; SD = 8.15)	For word-cued memories, a more pronounced bump was found between 5–19 years for AMs. For most important memories, a bump was found between 20–29 years in AM. Results suggested that the bump in most important AMs is a function of the cultural life script.
**38.**	Koppel, 2016 Denmark [89]	Comparing the distribution of fictional memories with the distribution of actual word-cued, and most important, AMs in a sample of 61–70 year-olds	Study 1: 42 participants, ages 61–70, mean age = 65.3, SD = 3.0 Study 2: 42 university students, ages 20–29, mean age = 23.3 years, SD = 2.2	A similarity was found between the temporal distributions of imagined memories and actual memories, suggesting the influence of constructive, schematic factors at retrieval on the bump.
**39.**	Krumhansl, 2013 USA [90]	Investigating whether the pattern of music-evoked AMs and preferences may have changed given the rapid evolution of popular music styles, and music’s prevalence, over the last few decades	62 participants (40 women, 22 men), mean age = 20.1 (SD = 1.30)	Showed the impact of music in childhood. An earlier peak occurred for 1960s music, which may be explained by its quality or its transmission through two generations.
**40.**	Leist, 2010 Germany [91]	Examining distributions of remembered negative and positive life events across the lifespan in a sample of adults in middle and old age	260 participants ages 41–86, mean age = 57.06 (SD = 8.06)	Distributions of positive, but not negative, life events showed a significant bump. There were substantial associations among number and valence of remembered life events, future time perspective, and functions of AM to create meaning, which remained significant after controlling for age and health.
**41.**	Platz, 2015 Germany [92]	Replicating findings based on a German sample and investigating the influence of the affective characteristics of the songs on the frequency of participants’ AMs	Experiment 1: 48 participants ages 52–82. Mean age = 67.1 (SD = 6.8) Experiment 2: 22 participants ages 60–74. Mean age = 66 (SD = 3.7)	Experiment 1 confirmed the bump from 15–24 years. Experiment 2 revealed that the affective ratings of songs were unequally distributed over the two-dimensional emotion space unlike the average rate of MEAMs which was nearly equally distributed.
**42.**	Rathbone, 2008 UK [93]	Exploring the relationship between memory accessibility and self with a novel methodology that uses the generation of self-images in the form of “I am” statements	Study 1: 16 participants ages 47–66 (11 women, 5 men). Mean age = 54.6 Study 2: 59 participants ages 39–76 (16 men, 43 women). Mean age = 53.95	Memories generated from “I am” cues clustered around the time of emergence for that particular self-image. Contrary to other memories, the first three selected memories showed a bump from 20–40 years.
**43.**	Rathbone, 2017 UK [94]	Investigating the role of the self in the reminiscence bump (heightened retrieval for events from young adulthood)	Study 1: 172 participants ages 40–80 (mean age = 49.97, SD = 8.92 Study 2: 151 participants ages 40–65 (30 men). Mean age = 46.98 (SD = 5.73)	The distributions of personally significant songs formed bumps, contrary to personally significant films. It was found that personally significant songs were more likely to be associated with episodic recollection compared to personally non-significant songs.
**44.**	Rubin, 1997 USA [15]	Analyzing the distribution of AMs across the adult life span	Six groups of 20 participants according to age: 20, 20b, 35, 70, 73, and 73b	There was a decrease in memories from the childhood years and a power-function retention for the most recent 10 years. Older subjects had an increase in the number of memories from the ages of 10–30.
**45.**	Rubin, 1997 USA [16]	Studying the distribution of AMs across the adult lifespan	Experiment 1: 20 undergraduates ages 20 years, I month and 20 years, 11 months (mean age = 20.37); and 20 older adults ages 70 years, 1 month and 70 years, 11 months (mean age = 70.34) Experiment 2: Five of the 70-year-olds, and 5 of the 20-year-olds from Experiment I	For word-cued AMs, older adults showed a bump from the ages 10–30. The five most important memories given by 20- and 35-year-olds were distributed similarly to their word-cued memories, but those given by 70-year-olds came mostly from the 20–30 year decade.
**46.**	Rubin, 1998 USA [11]	Reviewing AMs and preference literature to document that events or activities that occur between the ages of 10–30 are recalled more often and judged to be more important, or better, than events or activities from other age periods	60 participants tested in 1984: 30 university undergraduate students (mean age = 21.1, range 18–22 years), 30 older adults (mean age = 69.7, range 68–72 years) 60 participants tested in 1994: 30 university undergraduate students (mean age = 19.2, range 18–22 years), and 30 older adults (mean age = 69.8, range 66–72 years)	Factual, semantic, general-knowledge, and multiple-choice questions about the Academy Awards, the World Series, and current events from this period were answered more accurately by two different groups of 30 older adults tested 10 years apart.
**47.**	Rubin, 2003 Denmark [33]	Testing the life script explanation Examining the life script for the classes of events that Berntsen and Rubin used Examining classes of events that were used in Study 1	Study 1: 1,307 participants above the age of 16 (20–99 years). Age groups: 20–29, n = 234 (mean age = 24.84); 30–39, n = 270 (mean age = 34.56); 40–49, n = 255 (mean age = 44.00); 50–59, n = 205 (mean age = 54.43); 60–69, n = 158 (mean age = 64.32); 70–99, n = 186 (mean age = 78.03) Study 2: 87 undergraduate psychology majors ages 21–41 (68 women, 19 men; mean age = 26.2 years)	There was a bump for positive, but not negative, events, supporting the idea of culturally shared life scripts for positive, but not negative, events which structure retrieval processes and spaced practice.
**48.**	Rybash, 1999 USA [95]	Shedding light on how episodic memory and semantic memory contribute to older adults’ ability to retrieve autobiographical information across the different epochs of their lives	40 participants (mean age = 72.5; SD = 1.1)	Results showed a reminiscence bump from 6–15 years and a retention effect for both R (remembered) and K (knew) responses.
**49.**	Schrauf, 1998 USA [96]	Assessing the effect of a major cultural and linguistic transition, such as immigration, on AM recall, as well as the personal memories of persons who make such transitions, preferentially sampled according to language	12 participants from Spanish-speaking cultures who spent at least 30 years in an Anglo culture. 8 women, 4 men, ranging in age from 61–69 (mean age = 4.58; SD = 2.93).	An increase in memories followed the age of immigration and settlement. There were similar memory distributions for both the Spanish and English sessions. Events prior to migration were more frequently recalled in Spanish, whereas events after migration were more frequently recalled in English.
**50.**	Schrauf, 2001 USA [97]	Assessing autobiographical recall corresponding to the time of immigration	10 older participants who immigrated to the U.S. at ages 20–22, 24–28, and 34–35 (mean age = 64.5 years; SD = 2.5)	Instead of the usual reminiscence bump, an increase in autobiographical recall corresponding specifically to age at immigration was found. This may be due to: the encoding of novel events, and the “effort after meaning” required to integrate these events; followed by a relatively stable period (settlement) marked by release from proactive interference and spaced rehearsal.
**51.**	Schubert, 2016 Australia [98]	Investigating the utility of a non-SSP paradigm to determine whether the bump would emerge when participants were asked to recall a single memorable musical event from “a time long ago”	88 participants; 20–22 years old	Showed a bump as a result of participants spontaneously reporting the age at which they recalled a piece of music “from a time long ago,” with a significant bump occurring approximately 7 or 8 years earlier, at around 13 or 14 years old.
**52.**	Schuman, 2014 USA [17]	Comparing the memory when, for the first time, they are both obtained from the same large probability sample—in this case, from Americans aged 18 years and older	A large probability sample, N = 2,085	Findings revealed that there was peak in AMs and collective memories from 5–20 years and 5–30 years, respectively.
**53.**	Steiner, 2014 USA [99]	Examining the potential role played by mental representations of extended lifetime periods through a novel interview method	34 participants ages 59–92 (mean age = 73.06; SD = 8.5)	Older adults provided oral life stories; they divided their transcribed narratives into “chapters”. Participants’ ages at chapter beginnings and endings showed pronounced bumps between 17–24 years.
**54.**	Svob, 2012 Canada [100]	Investigating the intergenerational transmission of personal experiences and historically significant public events	60 undergraduates divided into two groups: the conflict group with 30 participants (14 men, 16 women; mean age = 18.8), and the non-conflict group with 30 participants (13 men, 17 women; mean age = 19.0)	Both groups produced sets of events that displayed a bump related to the parent’s estimated age at the time of the event. The findings suggest that transitional impact and perceived importance help determine which events children will remember from a parent’s life.
**55.**	Tekcan, 2012 Turkey [101]	Investigating age differences in life scripts by comparing adolescents, young adults, and older adults	98 participants (43 women, 50 men, 5 unreported): 51 young participants (41 women, 10 men), and 42 older participants (26 women, 16 men) Mean age: 14.04 for adolescents (SD = .20, age range 14–15), 20.61 for young adults (SD = 1.79, age range 18–29), and 78.45 for older adults (SD = 9.97, age range 54–96)	Results showed that adolescent and young adult scripts were more similar to each other than they were to older adult scripts. Older adult scripts were more typical, and showed a stronger bump for positive events corresponding to young adulthood. The bump for positive script events also emerged for events not experienced by the participants.
**56.**	Thomsen, 2008 Denmark [102]	Examining: (a) if cultural life script events structure recall of life story memories, (b) if chapters show a bump during young adulthood, (c) if start and end memories of chapters correspond to cultural life script events, (d) if memories referring to both cultural life script events and chapter starts or ends contribute more strongly to the bump than memories with no such reference	59 older participants: 27 women and 32 men with a mean age of 78.04 years (SD = 3.83; age range 71–88)	Chapter and life story memories showed a bump in terms of an increased recall of life story memories and chapters between ages 6–30. The bump was significantly stronger for memories that referred to both prominent cultural life script events and chapter start or end points.
**57.**	Thomsen, 2011 Denmark [103]	Testing the hypotheses that: (a) ages marking the beginning of positive, but not negative, chapters produce a bump, (b)that specific memories are over-represented at the beginning of chapters	92 participants, ages 49–75 (mean age = 59.58; SD = 6.49), with 67 women and 25 men	Only positive chapters formed a bump from 21–30 years, supporting the idea that chapters guide the search for specific memories, and that cultural life scripts contribute to the search process.
**58.**	Webster, 2007) Canada [104]	Examining reminiscence functions and vivid (i.e., landmark) personal memories in nine samples ranging from the teens to the nineties	198 participants ranging in age from 18–95 years	Older adults tended to reminisce more for social functions, while younger adults did more for self-functions. Older adults reported vivid memories that were less intimate and less negative. Adults of all ages showed the bump between 20–29 years.
**59.**	Wolf, 2016 Germany [105]	Examining individual differences in the distribution of word-cued AMs	118 older participants, mean age = 74.3 (SD 7.4 years), ranging from 60–99 years	Results showed that AM distributions indicated a bump between 10–20 years. Results agree with the life-story account for the bump which integrates central components of previous accounts.
**60.**	Maki, 2006 Japan [106]	Investigating if a reminiscence bump is found in AMs in Japanese elders, and the features and content of AMs in general, and in the bump	25 participants more than 60 years old	Results showed that although a reminiscence bump emerged between 7–25 years, memories within the bump did not differ from other memories in terms of rated features or content.
**61.**	Berntsen, 2011 USA [107]	Examining the effects of positive vs. negative emotion on the long-term accessibility of AMs Comparing the CES answered for a highly negative vs. highly positive event in a large sample of older adults	2,000 adults in their sixties	Overall, findings show that life script events expected to take place in young adulthood account for the greatest majority of all positive events, and explain the bump for positive events between 20–29 years—suggesting that many respondents used their cultural life scripts for guidance in nominating a “most positive” event.
**62.**	Raffard, 2010 France [108]	Examining specificity, meaning-making, content, narratives for coherence, and self-event connections Exploring the amount of experienced emotion during retrieval of SDMs	81 patients with schizophrenia and 50 healthy controls	Results suggest that schizophrenia patients have difficulty organizing and extracting meaning from their past experiences to create coherent personal narratives. Patients exhibited an early bump (15–19 years).
**63.**	Schlagman, 2009 UK [109]	Examining several memory characteristics (e.g., specificity, pleasantness, vividness) as a function of age and memory type Comparing the distribution of voluntary/ involuntary memories across the life span	44 younger, and 38 older, adults	Results showed that older adults report fewer involuntary and voluntary memories than younger adults. The life span distribution of involuntary and voluntary memories did not differ in young adults or older adults, and there was a bump between 10–30 years.
**64.**	Schroots, 2004 Netherlands [110]	Exploring the distribution of retrospective and prospective AM data across the lifespan, in particular, the bump	98 participants (47 men, 51 women)	Study results confirm the universality of the bump for older adults, as well as the recency effect, and showed a bump from 10–40 years.
**65.**	Raffard, 2009 France [111]	Investigating schizophrenic patients’ ability to recall self-defining memories, that is, memories that play an important role in building and maintaining the self-concept	20 inpatients and outpatients with schizophrenia	Patients with schizophrenia exhibited an abnormal reminiscence bump (15–19 years), and reported different thematic content (i.e., they recalled less memories about past achievements and more memories regarding hospitalization and stigmatization of illness).
**66.**	Cuervo-lombard, 2007 France [112]	Establishing that the defect of AM concern memories highly relevant to personal identity through the exploration of the reminiscence bump phenomenon in patients with schizophrenia	27 patients diagnosed with schizophrenia and 27 control participants	Patients diagnosed with schizophrenia recalled less specific memories than control groups and exhibited an earlier reminiscence bump. They recalled more public, and less private, events than control groups, and they gave fewer Remember responses. The reminiscence bump peaked from 16–25 years for patients and from 21–25 years for the control group.
**67.**	Bernsten, 2002 Denmark [113]	Examining the prevalence of involuntary memories across ages and the retention of positive and negative involuntary memories	1,241 participants between 20 and 93 years old	Older respondents exhibited a clear bump in their twenties for only the most important and happiest memories. Happy, involuntary memories were over twice as common as unhappy ones, and were the only memories showed a bump for the decade of the twenties.
**68.**	Maki, 2013 Japan [114]	Examining whether the temporal distribution of AM changes when different types of cue words are used to elicit memories, and how the type of cue word affects the phenomenal characteristics of the memories	76 participants, ranging in age from 21–69 years	Phenomenological properties of AMs cued by emotional and emotion-provoking words were rated higher than those of memories cued by neutral words. The peak in the temporal distributions of the AMs cued by neutral cue words was between 9–12 years, and for emotion-provoking words, 17–21 years.

Shows the data extracted and placed under appropriate sections corresponding to: author, year, country; study objective; sample size, and findings

1 Reminiscence bump, bump, or reminiscence effect, blip

2 Country = where the study was originally conducted. In case of missing information regarding country where study was conducted, corresponding author’s country is mentioned.

### Data synthesis

The researchers employed a narrative synthesis approach to abridge extracted study data. Narrative synthesis was deemed appropriate as it allows for both the synthesis of findings from several studies that use different research designs with varying characteristics of samples, and the application of an overall meaning to the data [115]. Through extraction, formulation (via a data extraction chart), and translation of the data into narrative summaries, the researchers utilized all accessible data. The initial stage of data familiarization occurred through the repeated systematic review of each research article. Formatting initial codes resulted in data refinement and the generation of themes from the data. Before allocating descriptive terms, themes were further refined so that the crux of all themes could be captured. These themes assisted in generating an analytic narrative during the final stage of report writing. Driven by the narrative synthesis approach, the data synthesis stage of this systematic review achieved the researchers’ goals of effectively drawing out—and giving meaning to—pertinent data from the research articles (please see S4 Table).

## Results

### Temporal and geographical representation of studies

The locations of the 68 studies varied: 19 in the U.S.; 9 in Denmark; 8 in the Netherlands; 7 in the United Kingdom; 4 in both Turkey and Japan; 3 in Germany, Canada and France, respectively; and 2 in Australia. One study took place in Bangladesh, Austria, Malaysia, and Trinidad and Tobago, respectively. Two studies included samples from more than one country: the first from Japan, Bangladesh, England, and China; and the second from the Netherlands and the United States (please see S1 Fig). Dates of published studies ranged from 1988 to 2017: 13 studies from 1988 to 1999, 29 studies from 2000 to 2010, and 26 studies from 2011 to 2017 (please see S2 Fig).

### Research study themes

**Fig 2:**
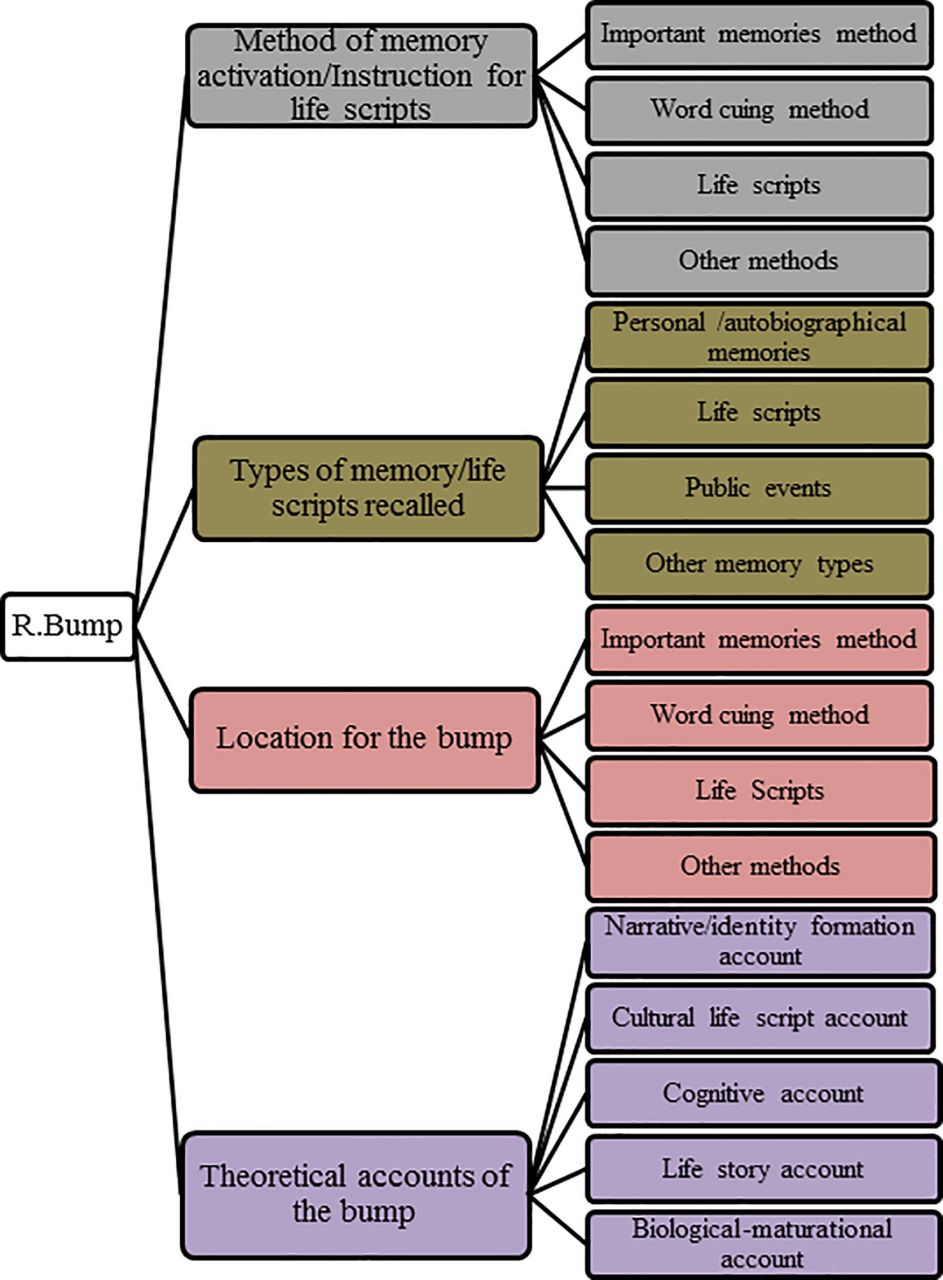
Summary of themes and sub-themes derived from these studies.

The articles selected for review comprised 68 quantitative studies (N = 68; see Fig 2).

Four main themes emerged after reviewing these studies on the reminiscence bump: (a) methods of memory activation/instruction for life scripts, (b) types of memory /life scripts recalled, (c) location of the reminiscence bump, and (c) theoretical accounts for the bump. These four themes evolved from 13 sub-themes.

#### Method of memory activation/Instruction for life scripts

The researchers used a variety of methods to recall memories or life events. Most studies implemented more than one recall method across a number of different populations.

**Important memories method.** Previous research consists of asking participants to report their important memories. Several studies ask participants about their most positive and negative, or most important and traumatic experiences, or important and surprising memories, used emotional cues (i.e. positive and negative), and asked participants to recall important positive and negative, or surprising positive and negative, events [22, 33, 35, 59, 72, 73, 100, 104, 107]. Furthermore, the past research studies focused on: (a) memories that are a vital component of life story, (b) life history timeline and significant life events narrative for the description of three events, (c) free narrative of life history about important life events and word cues, (d) descriptions of three self-defining memories, (e) Reminiscence Functions Scale and vivid memories that are important landmarks or turning points in life, (f) the Life Story Questionnaire for listing 15 personally important events or experiences, and (g) free narratives of life history; important life events [68–70, 108, 111, 113]. Two questionnaire-based studies instructed participants to report self-defining “I am” statements which were later used in recalling memories as well as a life event list for gathering distributions of positive and negative events [91, 93].

**Word cuing method.** A number of research studies use cuing methods for collecting memories, for instance odor cues, emotional cues and words cues. However, most of the studies used word cues for activating memories. The type of cues used, and their number, varies from study to study, and some studies use more than one cue recall method: (a) using word cues (i.e. emotional, emotion-provoking, and neutral); (b) instructing children to write future life stories using 10 word cues; (c) using both odor cues and word label cues; (d) reading novels and then implementing a cued recall task; (e) reporting important word-cue memories; (f) using 50 word cues for AMs; (g) employing 15 word cues pertaining to common locations, objects, positive emotions, negative emotions, and significant others; (h) Associative Memory Questionnaire with 18 word cues, (i) modified Autobiographical Memory Test having a list of 16 word cues; and (j) questionnaires implementing both the word cues and important memories method [17, 60–62, 64, 88, 89, 96, 114, 116].

Several studies assess life events through: (a) the use of various emotional cues to elicit and record events in the course of a typical and hypothetical person, and (b) verbal reporting of events from personal lives and diaries. Cued recall methods include: 10 word cues, 40 word cues for obtaining specific memories, and 20 ambiguous and 20 unambiguous single names [24, 75, 109]. A few studies use the Galton-Crovitz cueing method and Robinson word cuing technique [30, 76–78, 81, 82, 86, 105, 106]. The number of word cues used to elicit memories range from 15–124 [15, 16, 74].

**Life Scripts.** The studies on life scripts used various instructions to explore the reminiscence bump. For instance, these studies assessed the reminiscence bump through: (a) asking participants to report important events likely to occur in the life of a newborn or an elderly person; (b) the life script questionnaire; and (c) the expected timing of the public event [32, 71, 84, 87, 101].

**Other methods.** Other memory recall methods that were not associated with the three methods already discussed, were found in some studies (e.g., dream diaries, the evaluation of participants’ reactions to nostalgic advertising, a modified version of timeline methods for recording specific memories, issues surrounding the Academy Awards, World Series, and current events, life regrets’ content and chronology, and free recall events having public or private nature[9–11, 63, 65–67, 85, 110]. Adults were interviewed to discover their: recounted and oral life stories; specific autobiographical lifetime events; three favorite books, movies, and records, or the five best football players; and memories using the Yearly News Memory Test (YNMT) comprising 30 open-ended and multiple-choice questions [79, 80, 83, 97, 99, 102, 103, 112]. A few studies used music clips and instructed participants to imagine music as a means to access associated memories [90, 92, 94, 98].

#### Types of memory/life scripts recalled

The 68 research studies elicited a variety of responses in types of memories activated.

**Personal /autobiographical memories.** One study elicited personal future life stories of children, and events from those stories [60]. In studies where participants reported autobiographical events for various types of cues, retrieved memories were from personal pasts [15, 16, 30, 33, 59, 61–63, 69,74, 75, 76–78, 81, 86, 89, 90, 92, 93, 96, 98, 104–106, 109, 114]. The following were elicited in various studies: personal memories of adults spanning their life course, meaningful life events, life stories from across participants' life spans, lists of life events for AMs and collective memories, life-lines for both past and future events, and self-defining AMs [17, 22, 35, 66, 70, 72, 73, 91, 95, 97, 99, 102, 103, 107, 108, 110–113]. One study collected participants’ personal specific memories and specific memories related to 70- and 80-year-old hypothetical cases [68].

**Life scripts.** These studies asked participants about important events likely to occur in the life of a newborn and important events a newborn or an elderly person would experience during his/her lifetime [32, 71]. A few research studies investigated original and modified versions of the life script questionnaire, and probed cultural expectations for the expected timing of the public event [84, 87, 101].

**Public events.** A number of research studies focused on memories for important public and private news items, as well as public events. [10, 80, 81]. In another study, authors elicited both autobiographical and public events [88].

**Other memory types.** Other research studies focused on curves of forgetting after memorizing a 10-chapter autobiographical novel, flashbulb memories, AM recall, and memories from the lives of participants’ parents [24, 64]. Some studies investigated dreams of older adults’ life regrets; flashbulb memories; reactions to nostalgic advertisements; and responses to the World Series, academy awards, and current events [9, 11, 65, 67, 85]. And in other studies, researchers asked participants to report three favorite books, movies, and records as well as the five best sports players of all time [79, 83, 94, 100].

#### Location of the bump

The temporal range of the bump varied along with the method of memory activation and presence or absence of a mental health issue (see Table 2).

**Table 2:** Method of memory activation/life scripts and bump range.

Serial no.	First Author/Year	Method of memory activation/Instruction for life scripts*	Range of bump
**Important memories method**			
1	Berntsen, 2011 [107]	Participants recalled the most positive event of their lives and the traumatic or negative event that currently troubles them the most	Most positive event of lives: 20–29
2	Dickson, 2011 [68]	In Study 2, participants recalled an especially positive event and an especially negative event, or a surprising positive event and a surprising negative event In Study 4, participants recalled a highly expected event and a highly unexpected event. Participants also rated the emotional valence of both the highly expected and highly unexpected event they mentioned Though the authors subsequently report the individual distributions for both the positive and negative expected and unexpected events, they also report the overall distributions, collapsed across positive and negative events; we report the bump in these overall distributions.	Especially Positive Event: 16–30 Surprising Positive Event: 16–30 Highly Expected Event: 16–30 Highly Unexpected Event: 16–30
3	Ece, 2014 [69]	Life script task: ten most important events in an expected life course of a person in their culture Autobiographical memory task: report 10 important personally experienced life events	Expected life events: 16–20, 21–25, and 26–30 years Autobiographical memories: no reminiscence bump but clear childhood amnesia and recency effects
4	Fromholt, 2003 [73]	Centenarians provided autobiographical memories to either a request for a life narrative or a request to produce AM to 15 word cues. Experiment 1: 15-minute free narrative of life history following the instruction: “Tell about the events that have been important in your life” Experiment 2: provide a specific, datable auto-biographical memory for 15 word cues (English translation: cat, cotton, fire, flag, flower, friend, money, morning, nail, picture, road, storm, sugar, ticket, and window).	15–30
5	Rubin, 2003 [33]	Study 1: Memories for most afraid, most proud, most jealous, most in love, most angry, and most important event and whether this event was positive or negative Study 2: Estimate 70-year-old men or women person’s age during each of a series of emotionally charged events	Study 1: • Most positive important event: peaks in 20s • Most angry: 13–19 • Most afraid: 20–29 Study 2: • Most in love, Most important: 20–29 • Happiest, Proudest: 30–39
6	Svob, 2012 [100]	Participants recalled and dated 10 important events from one of their parents’ lives	Reminiscence bump related to parents’ age at the time of the recalled events = 20–30 Mini-bump from the time the participant was born up to the present = 40–50
7	Berntsen, 2002 [31]	Participants were asked age of happiest, saddest, most traumatic, most important memory, and most recent involuntary memory	Most important event: 20–29 Happiest event: 20–29
8	Leist, 2010 [91]	Life Events lists: Participants marked each negative and positive life event on the lists, if the event occurred Functions of autobiographical memory, Time and Future Perspective Questionnaire	20–29
9	Rathbone, 2008 [93]	All memories participants generated in association to “I am” statements: • Study 1: 10 memories to each of three statements • Study 2: 8 memories to each of four statements, as well as just the first three memories	All Memories: No Bump First Three Memories: 20–40
10	Fromholt, 1991 [72]	15-minute free narrative of life history focusing on important events 10-item meta-memory questionnaire	10–30
11	Elnick, 1999 [70]	A life history timeline and a description of three significant life events narrative The Family APGAR Bloom's Colorado Self-Report Measure of Family Functioning Defense Styles Questionnaire Modification of the Close Relationship Questionnaire Self-Representations	Life history timeline: 20–29 Three significant life events narrative: 20–29
12	Fitzgerald, 1996 [35]	Participants recalled four events that they would include in a book about their life	16–25
13	Glück, 2007 [22]	Participants completed the Life Story Questionnaire, in which individuals list up to 15 events or experiences that they consider most personally important in their life	16–30
14	Alea, 2014 [59]	10 word cues: paper, pencil, child, hostage, seat, hospital, corpse, anxiety, candy, hammer Life events questionnaire for 14 life events	Positive Memories: 6–15; 26–30 Negative Memories: 6–15; 26–30
**Word and other cuing methods**			
15	Bohn, 2011 [60]	Study 1: children wrote future life stories Study 2: word cues to write down events from their future lives	Study 1: • Life scripted events: 20–30 • Non-life scripted events: 20–30 Study 2: Life scripted events: 20–30
16	Chu, 2000 [61]	27 odor-related words	11–25
17	Conway, 1999 [62]	Young and older groups of Bangladeshi participants recalled and dated autobiographical memories from across the lifespan in response to 15 word cues	10–30 Older adults 2^nd^ bump: 35–55
18	Copeland, 2009 [64]	Forgetting curves for information read in a 10-chapter novel where each chapter covered an approximately 10-year period in the life of the protagonist	Novel summaries: 20s, 50s Cued protagonist task: • Chapter 3: 22 years • Chapter 6: 50s
19	Koppel, 2016a [88]	Word cuing method (10 word cues; i.e. money, water, child, clothing, church, woman, street, fire, kiss and city) Important memories/important events method for three most important memories	Cue word method: 5–19 Important memories method: 20–29
20	Koppel, 2016b [89]	Study 1: Seven word-cued and important autobiographical memories Study 2: Seven word-cued and important autobiographical memories of a hypothetical 70-year-old	Study 1: • Actual cue word method: 6–10 • Actual important memories method: 16–30 Study 2: • Imagined cue word method: 6–25 • Imagined important memories method: 16–30
21	Maki, 2013 [114]	22 word cues (emotional, emotion-provoking, and neutral)	Neutral cue words: 9–12 Emotion-provoking words: 17–21 Emotional cue words: No peak (8–30)
22	Rybash, 1999 [95]	18 Total words, with an unspecified combination of nouns, activity verbs, and affect terms Authors collected remember/know ratings for each memory, and plotted the distributions separately for memories that received either remember, or know judgements	6–15
23	Schrauf, 1998 [96]	Autobiographical memories to 50 word cues	10–30, 20–24, 34–35
24	Schuman, 2014 [17]	A specific event from own life in response to eight word cues (e.g. flower, horse, fire, bird, lake, window, book, and friend) Collective memories for national and world events rated important	Autobiographical memories: 5–20 Collective memories: 5–30
25	Fitzgerald, 1988 [24]	Study 1: 40 word cues autobiographical memory task Study 2: three flashbulb vivid memory descriptions	16–20
26	Haque, 2010 [75]	Participants recalled the happiest event, saddest event, most important event, most traumatic event, most angry event, most in love event, most jealous event, most proud event, most fearful event, the event indicating the highest success, and the most surprising event of their lives	Happiest Event: 20–29 Most Important Event: 20–29 Most in Love Event: 20–29
27	Jansari, 1996 [76]	Experiment 1: participants recalled events freely or under instructions to avoid recent memories. (16 Nouns, 16 Activity Verbs, and 16 Affect Words) Experiment 2: recall was constrained to particular life periods. (Participants recalled memory to sixteen word cues (6 nouns, 5 verbs, and 5 affect words) from)	6–15
28	Janssen, 2003 [77]	64 word cues	10–30
29	Janssen, 2005 [78]	10 word cues: recall and date autobiographical memories	15–25 Peaks: Men, 15–18; Women, 13–14
30	Janssen, 2008 [30]	10 word cues: describe the personal events that came to mind	6–19 (Overall) Young adults: 8–12 Middle-aged adults: 6–16 Older adults: 7–20
31	Janssen, 2011 [81]	Galton–Crovitz test: 64 word cues	4–21
32	Janssen, 2011 [82]	10 noun word cues	6–20
33	Kawasaki, 2011 [86]	Each participant received a random selection of 10 word cues out of 64 word cues	Young adults: 5–13 Middle-aged adults: 6–15
34	Rubin, 1997 [15]	124 word cues 5 most important memories	10–29
35	Rubin, 1997 [16]	124 word cues for autobiographical memories	10–30
36	Wolf, 2016 [105]	39 word cues: nouns, verbs, and adjectives	10–20
37	Maki, 2006 [106]	9 nouns, 7 emotional words, and 6 emotion-provoking words	7–25
38	Schlagman, 2009 [109]	Diary Word cues (e.g. cabin, pipe, elephant, chest, silk, theatre, watch, whip, pillow, and giant)	10–30
39	Gidron, 2007 [74]	Modified Autobiographical Memory Assessment: autobiographical memories to 4 groups of 16 word cues reflecting events from childhood, adolescence, adulthood and late age	10–25
40	Raffard, 2010 [108]	Self-defining memories questionnaire: Participants recalled three self-defining memories	Control: 20–24 Schizophrenia: 15–19
41	Raffard, 2009 [111]	Self-defining memories questionnaire: Participants recalled three self-defining memories	Schizophrenia: 15–19 Control: 20–24
42	Webster, 2007 [104]	Described a vivid memory that “was important in your life, or that changed how you think about yourself”	20–29
**Life Scripts**			
43	Berntsen, 2004 [32]	Reanalysis of earlier studies on age norms How old hypothetical centenarians were when they were most happy, most sad, most afraid, most in love, and had their most important and most traumatic experiences Seven important events likely to occur in the life of a newborn	Positive events: 15–30
44	Erdoğan, 2008 [71]	List the seven most important events a newborn or an elderly person would experience during his/her lifetime and estimate the prevalence, importance, age-at-event and emotional valence of each	Study 1: • Positive events: 20–30 • Negative events: 10–20 Study 2: Positive events: 20–30
45	Janssen, 2015 [84]	Life script questionnaire (i.e., personal events; seven most important events expected to happen in a prototypical infant's life) Modified version of the life script questionnaire (i.e., public events)	16–30
46	Koppel, 2014 [87]	Study 1: probing cultural expectations for the expected timing of the public event that a typical person considers to be the most important of their lifetime Study 2: probing cultural expectations for the expected timing of the objectively most important public event of a typical person’s lifetime	Study 1: 11–30 Study 2: 11–30 Peak: 16–20, 21–25
47	Tekcan, 2012 [101]	Adolescents, young adults, and older adults listed the seven most important events that a typical newborn would experience in a lifetime	Positive events: 30–39 Negative events for adolescents, young adults: 13–19
**Other methods**			
48	Cappeliez, 2008 [9]	Write in home dream diary for a week	15–25
49	Conway, 2005 [63]	Participants were required to recall 20 specific AMs from their own lives	10–30
50	Davison, 2008 [65]	A questionnaire in which 40-year-olds and senior adults described and dated up to five regrets for specific or general experiences	Study 1: 10–19 Study 2, Participants in Their 40s: 20–29 Study 2, Participants in Their 60s: 20–29
51	Demiray, 2009 [66]	Participants free-recalled autobiographical memories, and were given 7 min to retrieve as many memories as they could for each five-year interval in their life (e.g. from 20–25)	10–30
52	Denver, 2010 [67]	Free recall flashbulb memories from personal lives, 9/11, and a personal flashbulb memory. Adapted version of the Memory Characteristics Questionnaire	Study 1: 10–30 Study 2: 10–30
53	Holmes, 1999 [10]	Participants free-recalled when they had learned public and private items of news	Public Items: 10–19 Private items of news: 20–29
54	Rubin, 1998 [11]	Factual, semantic, general-knowledge, multiple-choice questions about the Academy Awards, the World Series, and current events	10–30
55	Schroots, 2004 [110]	Life-line Interview Method: Participants were asked to draw a life-line for both past and future events, and to date and label each event	10–40
56	Cuervo-lombard, 2007 [112]	Participants recalled 20 specific autobiographical events during their lifetime	Patients: 16–25 Controls: 21–25
57	Schrauf, 2001 [97]	Hispanic adults who immigrated to the USA at ages 20–22, 24–28, and 34–35 narrated their life‐stories on twice, once in English and once in Spanish	Early immigrators (ages 20, 21, 22) = 20–29 Middle immigrators (ages 24, 26, 28, 30) = 30–39 Late immigrators (ages 34, 35, 35) = 40–49
58	Steiner, 2014 [99]	Novel interview: older adults provided oral life stories, and they divided their transcribed narratives into chapters	17–24
59	Thomsen, 2008 [102]	Participants recalled the five events that they considered most central to their life story	6–30
60	Thomsen, 2011 [103]	After dividing their life story into chapters, participants recalled an important specific memory from their most positive and most negative chapter, respectively	Memory from the most positive chapter: 21–30
61	Krumhansl, 2013 [90]	Young adults’ personal memories associated with top music hits over 5-and-a-half decades	Unclear
62	Platz, 2015 [92]	Experiment 1: Participants listened to excerpts from 80 number-one, popular music hits from 1930 to 2010 and gave written self-reports on music-evoked autobiographical memories Experiment 2: Other participants rated affective characteristics	15–24
63	Rathbone, 2017 [94]	Memories related to top-grossing films and songs, selecting the five that were most personally significant	Study 1: Personally significant films and songs, Songs: 15–19 Study 2: Songs, 10–14 Films: No Bump
64	Schubert, 2016 [98]	Participants were asked to recall a single memorable musical event from “a time long ago”	13–14
65	Janssen, 2007 [79]	Participants were asked to name their three favorite books, movies, and records and time period they first encountered them	16–20
66	Janssen, 2008 [80]	Yearly News Memory Test (YNMT)	10–25
67	Janssen, 2012 [83]	Participants were asked who they thought the five best players of all time were	11–30
68	Ju, 2016 [85]	Reactions to nostalgic advertising	15–24

* For the ease of categorization, we have classified studies using more than one method of memory activation/instruction for life scripts under one method based upon the dominant method employed. The “Range of bump” column, however, illustrates ranges for all the methods utilized within each study despite the study’s location under the heading for the dominant method employed.

The range and location of the bump vary with respect to the method for activating different types of memories.

**Important memories method.** Research studies using this method revealed the location of the bump to be from a minimum of 10 years to a maximum of 40 years of age, and some studies also showed a more localized peak from ages 16 to 20 years of age [33, 35, 59, 68, 93, 100, 104, 107, 113]. Findings of another study did not show the bump for AM distribution, however, a bump appeared for the life script distribution as a result of suppressing typical life events [69]. Three age intervals corresponded to the bump period (i.e. 16–20, 21–25, and 26–30 years) [69]. The responses to a newborn questionnaire demonstrated a considerably large bump for positive events in the third decade of life, a very small bump for negative events in the second decade of life, and a clear bump for positive events between 10 to 30 years of age [71]. The range of the bumps for positive, negative, expected, unexpected events, and significant life events narrative were between 16 to 30 years and 20–29 years of ages, respectively [22, 70, 72, 73, 91]. A few studies compared the bumps of control groups and patients with schizophrenia and found different results for both groups: 15–19 and 16–25 years for patients with schizophrenia; 20–24 and 21–25 years for control groups [108, 111].

**Word cuing method.** Using these methods, the distribution of the future life stories of Danish children illustrated a bump in young adulthood, and this bump consisted of life-script events [60]. Studies discovered bumps from a minimum of 6 years of age to a maximum of 35 years of age [11, 15, 16, 24, 30, 61, 62, 74–76, 78, 81, 82, 88, 89, 95, 106, 109, 114]. One study, using a novel cuing method, revealed two bumps: The first bump appeared when the protagonist of the story was 22 years old, and the second occurred when she was in her 50s and undergoing significant life changes [64]. Studies revealed the timing of the bump to be between the ages of 10 to 30, with peaks occurring between 15–18 for men, 13–14 for women, 5–13 for Japanese adults, and from 5–30 for personal and collective memories; a peak in recall occurred between 10–19 years of age for public items and 20–29 years of age for private items, and a bump between 10 to 30 years of age [17, 77, 78, 86, 105].

**Life scripts.** The studies on life scripts revealed varying locations of the bump. These studies revealed the bumps of positive and negative events from a minimum of 6 to a maximum of 39 years of age [32, 71, 84, 87, 101].

**Other methods.** A study using a heterogeneous sample from five countries (i.e. Japan, Bangladesh, U.K., China and the U.S.) revealed that more than 50% of memories were recalled from the ages of 10–30 [63]. Studies also showed a bump in late adolescence to early adulthood as well as a bump between 15 to 24 years of age [9, 11, 65, 67, 79, 85, 92, 94, 99]. Two studies, using a life history timeline method and a “life-line” interview method, discovered the bump between 10–30 and 10–40 years of age, respectively [66, 110]. A study using cues explored memories of younger and older Bangladeshi individuals: Younger adults showed a bump between 10–30 years of age, whereas an older group revealed a second bump between 35–55 years of age [62]. The bump corresponding to ages of immigrants at the time of immigration, occurred between 6–30 and 10–25 years of age [80, 97, 98, 102, 103]. A study using free recall flashbulb memories from personal lives reported a bump between 10 to 30 years of age [67]. A study conducted on patients with schizophrenia revealed bump between 16–25 years for patients and 21–25 for controls [112].

#### Theoretical accounts for the bump

There are a variety of theoretical accounts for the bump, and each account has received varied levels of support in the research. The narrative/identity account is fully supported by the findings of seven studies [9, 10, 35, 36, 62, 70, 93], and partially supported by the findings of six studies [24, 72, 76, 108, 111, 112]. Two studies illustrated complete support for the cultural life script account [33, 71], while ten studies showed partial support [32, 60, 75, 87, 89, 100–103, 114], and two studies demonstrated no support at all [59, 84].

Four studies supported the “life story” account [22, 66, 73, 105], three studies supported differential encoding and differential sampling [78, 79, 92], one study supported the “life-span perspective” [110], one study supported the cognitive account [97], and two studies showed some support for the “biological-maturational” account [30, 74]. Several research studies supported more than one theoretical account for the bump: the cultural life script and “novelty” accounts [64]; the narrative/identity and cultural life script accounts [65, 68, 107]; the narrative/identity, cognitive and “maturational” accounts [67, 96, 109]; the cognitive, narrative/identity, cultural life script, and life story accounts [69, 94]; the cognitive, narrative/identity, and cultural life script accounts [80]; the biological, narrative/identity, and cultural life script accounts [83]; and the cognitive and narrative/identity accounts [11].

## Discussion

Despite the wealth of evidence existing on aspects of the reminiscence bump that present when using different methods for activating memories, there is a limited understanding of which theoretical account—or accounts—offers the best explanation for the bump, and the reasons for variation in the exact location of the bump. The aim of this systematic review is to establish a current evidence base concerning the understanding and formation of the bump, and to add to the existing body of literature on AMs, other kinds of memories and the reminiscence bump.

### Summary of study findings

The results of this systematic review on the reminiscence bump are based on the analysis of 68 selected studies retrieved from 9 scientific databases and screened by the researchers. The results reveal that methods for activating memories/instruction for life scripts include the important memories method [33, 69, 100], word cuing method [17, 60, 61, 64, 68, 114], life scripts [32, 71, 84, 87, 101], and other heterogeneous methods [72, 73, 79, 80, 83, 97, 99, 102, 103]. A variety of responses were elicited from the participants including: AMs [15, 16, 30, 33, 59, 61–63, 74, 76–78, 81, 86, 90, 92, 93, 96, 98, 104–106, 114], memories for public events [10, 80, 81], life scripts [32, 71, 84, 87, 101], and other heterogeneous responses [79, 83, 94, 100].

The exact location of the bump varied with each method for activating memories. For instance, with the important memories method, studies showed the bump between 10–30 years of age [11, 22, 32, 33, 35, 65, 67, 69, 71, 84, 87, 93, 95, 100]. For word cuing method, the bump began as early as 5 years, and lasted until as late as 30 years of age [10, 17, 77, 78, 86, 114]. Likewise, for the studies using life scripts, the location of the bump was from 6 to 39 years. Also, there are a variety of theoretical accounts offering an explanation for the bump for different kinds of memories activated, such as the narrative/identity account which received significant support from eight studies [9, 10, 24, 35, 63, 70, 93, 94], the life story account garnering sound support in four studies [22, 66, 73, 105], and the cultural life script account finding substantial support in two studies [33, 71]. The narrative/identity account received partial support from seven studies [10, 24, 72, 76, 108, 111, 112], and the cultural life script account received a degree of support from ten studies [32, 60, 75, 87, 89, 100–103, 114].

### Interpretation of study findings

Past research indicates that the cues used to induce the memories influence both the proportion of memories recalled and the location of the bump [8, 15]. A variety of cuing methods exist, therefore different retrieval processes help to explain the differences in reported location of the bump and the distribution of AMs [117]. The research demonstrates that word cues initiate an associative, “bottom-up” process in memory, whereas the important memories method prompts a strategic, “top-down” process in memory that is organized around important memories [8, 12]. The Attention-to-Memory hypothesis [118, 119], proposes that the two major brain regions playing different roles in attention are the dorsal parietal cortex and the ventral parietal cortex. The dorsal parietal cortex is associated with top-down attention (i.e. selecting stimuli on the basis of the internal goals of the individual); the ventral parietal cortex is concerned with bottom-up attention (i.e. permitting the detection of related stimuli) [120, 121].

The Attention-to-Memory hypothesis states that in addition to playing a significant role in attention, the two cortexes play similar roles in memory retrieval [122]. The dorsal parietal cortex initiates the assignment of attentional resources towards retrieval of a specific memory (i.e. top-down Attention-to-Memory) [122]. The important memories method supports this, as the dorsal parietal cortex initiates top-down attention when retrieval relies on memory search [8, 12]. Alternatively, in word cuing methods, the ventral parietal cortex initiates a bottom-up attention focus on the basis of retrieved content. Recent reviews on recognition memory studies support these theories and the localization of the top-down and bottom-up attention [123]. The instructions for the important memories method initiate a search for relevant memories—a role of the dorsal parietal cortex. The instructions for memories related to word cues initiate rapid detection of memory content—a role of the ventral parietal cortex.

Memory activation methods play a significant role in the nature of the memories activated. Word cuing methods yield unbiased sampling of memories across the entire life span [12], whereas the important memories method focuses on eliciting the most important memories of a person’s life, and tends to produce a narrative-based search [8, 12]. Important and self-defining memories are closely linked to the meaning-making processes of individuals [124, 125]. The different types of memory activation methods and theoretical accounts of the bump have common underlying mechanisms influencing bump location. The range and location of the bump vary according to memory activation method: word cuing methods yield a disproportionately large number of recent memories and an earlier bump location (see Table 2), as well as with respect to the type of memories activated. Differing locations of the bump have significant implications for theoretical accounts explaining the bump [8]. In this review, the researchers present a general range of the bump approximated through the analysis of all studies: 16–30 years of age for the important memories method; 5–30 years of age for word cuing methods. A past review paper calculated the mean range and midpoint of the bump formed through different cuing methods. The mean range of bump for word cued and important memories was calculated between 8.7 to 22.5 and 15.1 to 27.9 years of age, respectively [8]. The differences in location of the bump could be due to different methods of activating memories or differences in memory types.

Disparate bump ranges are indicative of the processes occurring at retrieval, favoring a retrieval-based account of the bump. This contradicts the accounts focusing on characteristics of the memories themselves (i.e. narrative/identity account and cognitive account) [11, 24, 25, 35, 40], or the effectiveness of encoding during the bump period (i.e. cognitive abilities account) [11, 126]. These findings suggest a schema-based explanation of the bump (i.e. cultural life script account) rather than an individualistic and memory-based account [32]. There is considerable supporting evidence for the role of cultural life scripts in organizing the retrieval of AMs for important and emotional events [32, 33, 68, 125, 127]. According to Rubin (2015), variations in bump peaks cannot be explained solely in terms of encoding, or by theoretical accounts, especially those that consider adolescence and early adulthood periods to be when the emergence of identity or heightened cognitive ability occurs [3, 11, 117]. Different methods of activating memories give rise to different bumps and the mechanism underlying the bumps for different types of memories are different. The mechanism is different when other kinds of responses or memories are elicited (e.g., life scripts, dreams, etc.)

Life scripts allow encoding and rehearsal of an event by attributing the personal events to some culturally shared importance. A majority of the life script events are anticipated, prepared for, and given a certain meaning before they even happen in a person’s life [32]. According to established empirical evidence, the recall of important life events is structured by the life scripts, however, there is no such structuring of life events through cues, as cues are not likely to initiate culturally shared schemata for important transitional events [46]. The cultural life script account is possibly the reason behind the prevalence of important memories in the bump and a greater proportion of life script events in important memories.

This review demonstrates that the cultural life script account received considerable support, yet accepting it as a possible explanation for the bump would be problematic owing to some inherent flaws. The cultural life script account utilizes the concept of life scripts—cultural expectations about the timing and arrangement of significant transitional life events in a prototypical life course—to provide a cultural explanation for the bump [31–33]. The empirical evidence partially supporting the cultural life script account also employed other theoretical accounts to explain these findings [32, 60, 75, 87, 89, 100–103, 114]. Very few studies have compared the life script and real life events [68, 75]. Thus, there is a lack of evidence for comparison of the bump patterns for life script events and real life events, and the similarity between the cultural life script events and non-scripted events [33, 60, 68, 128]. The similar bump patterns for both life script events and real life stories, run contrary to the premise of the cultural life script account, meaning that the cultural life script is not solely responsible for the recall of the events and formation of the bump [32].

The fact that the bump is not based on the age of the memories, but on the age of the person recalling at the time of encoding, implies that findings of an artifactual, retrieval based account of the bump (i.e. the cultural life script account) can be rejected [3]. Memories may be easily retrieved due to the originality of the experiences (i.e. high emotional valence), or because they play a role in higher-order structures of the personality (i.e. significance or self-relevance). A bump for vivid memories may occur due to salience rather than mere nostalgia, but are significant because they define who a person is [24]. The ability of a person to consciously recall memories, to identify them as linked to his/her personal past, and to relate them to his/her goals and desires permits the formation of a coherent personal narrative concerned with the present and the future [111, 129].

Researchers propose that there is an emergence of adult identity during late adolescence and early adulthood [130]. This period may contain many self-defining incidents which link the self of an individual to that particular reality [131]. Therefore, an account focusing on the role of self in the bump (e.g. narrative/identity account) can be used to explain the occurrence of memories from this period [62]. The bump reveals an era in an individual’s life that is crucial for the development and maintenance of a stable self, as basic cognitive changes across the life span cannot be solely responsible for shaping retrieval [11, 130]. It is likely that the development of a new self initiates preferential encoding due to the importance of the formation of particular personal and cultural identities [22, 31, 132]. As AMs ground the self, there is a possibility that the importance of identity development stimulates the use of cognitive mechanisms [11, 36].

The narrative/identity account received significant support: a study analyzing dream content, temporally linked with the bump, revealed themes associated with identity and life goals [9]. However, the dreams were collected from professional career women mostly at retirement age, who were already experiencing a transition—during which most people are concerned with life orientation and purpose—that could trigger the importance of identity. Another study collected benchmarked memories from the life history timeline, revealing a bump associated with identity formation in early adulthood, however, the bump was seen concerning only family or relationships [70]. A high correlation between levels of rehearsal and preoccupation with stories from participants’ lives could show potential support for the narrative/identity account, but causal claims cannot yet be made [35].

Free recall of public and private news items revealed differential bumps: 10 to 19 years for public items and 20 to 29 years for private items [42]. The earlier bump reflects a period of formation of generation identity, while the later bump mirrors a period of formation of intimate relationships [42]. The generation of self-images in the form of “I am” statements to test the relationship between memory accessibility and self, lends support to the narrative/identity account due to the clustering of AMs around the time of self-formation [93]. However, this evidence was only found when the first three memories representing each self were compared at age 20 versus age 40. Another study revealed an absence of the bump for AMs when highly self-relevant life events were supressed [69].

Similar support is shown in studies by examining the relationship of highly positive and highly negative events with life story and identity [107]; and the role of generational identity with the development of an integrative self behind the bump [62]. The latter study demonstrates an accessibility of AMs from a period outside of the reminiscence bump that are suggested to be relevant to the self [111]. In this study, a group of older Bangladeshi participants presented a second bump for the ages of 35 to 55 years, coinciding with the period of Bangladesh’s war for independence in 1971. It is suggested that the second bump is due to the enhanced retrieval of AMs from a period when Bengalis, as a nation, were struggling to establish their own independent country, and to uphold their collective Bengali identity. However, a current debate exists on whether individuals recall public events from the bump period because of their identification with those events, or because of better encoding of them [80]. The narrative/identity account states that all the memories for adolescent events are not necessarily self-narrative memories, but rather that more events from this phase, with a better availability for recall, form a pool for self-narrative memories.

Alternatively, the ratings of re-living and vividness showed no differences between bump and non-bump memories, thus rejecting the role of phenomenological features of memories in the bump formation [82]. One study investigating the personal significance of songs showed a bump for both R (remember) and K (know) ratings [94]. Although a greater number of personally significant songs were associated with R ratings, K ratings also formed a bump, even though according to the narrative/identity account, they should not do so. The question of circularity raised in the research has not been answered (i.e. whether the selection of songs as personally significant is due to their association with particular memories, or high personal importance of a song leads a person to relate this song to specific memories from the time it was heard) [94].

The preponderance of memories from the bump period does not mean that these memories are related to identity formation unless there is a direct retrieval of identity related memories and an analysis of the lifespan distribution of these memories is performed. Although the narrative/identity account states that the bump is the result of an identity-relevant process, substantial evidence is still needed. Furthermore, the way identity-related questions are formulated, and the functional demands of answering these questions for the participants, impacts the construction of available memories. Various factors such as emotional salience, specific temporal and geographical context, sociocultural factors and a self-reference effect might influence the preferential retrieval of personally significant, over non-significant, events [133, 134]. As a result of the interplay between these factors, information associated with the self tends to be remembered best [135]; and thus, these more easily remembered memories may not necessarily indicate that bump memories are from important identity-forming events.

The heterogeneous methodologies of the studies in this review have created difficulty for the authors in discerning a link between identity formation and the bump, as the studies are based on memories, or important memories, and ratings. A major issue with ratings lies in the fact that these ratings reflect how study participants currently feel about their experiences rather than what they felt at the time of encoding the memories. Therefore, ratings have a limited role in identity related explanations of the bump (e.g. it is difficult to ascertain how study participants recalled and reported their judgments of rehearsal to reflect true/precise rehearsal rates).

The authors did not find any direct request to recall specific self-defining memories (SDMs) in the research, while important life event narratives were requested. Two studies investigated the role of the self in AMs by examining SDMs [136]. The exploration of SDMs is an important approach for understanding the association between identity and the bump as they are memories of events that one draws on to inform one’s sense of identity [137, 138]. A few studies did investigate the self-relevance of autobiographical events in the bump and their centrality to the life story and identity, SDMs, and self-images [93, 107, 108, 111]. The identity of individuals depends upon their ability to recall personal history, in the form of self-defining memories [139]. Therefore, there is a need to look deeper into the encoding and retrieval of event-specific temporal knowledge for understanding the self and identity [140].

The key to understanding the bump may lie in the memories of self-defining events during adolescence and early adulthood, as the narrative/identity account claims that many memories found in the bump are from this period [24, 93, 131, 141]. These memories play a vital role in the regulation of mood and direct functions of the self [142, 143]. Studies using measures for memory function and the self asked participants to report 20 “I am” self-concepts, thereby collecting concepts/roles significant to their definitions of self [39, 93, 144, 145].

### Theoretical implications

The present systematic review extends the body of knowledge on the reminiscence bump, highlights theoretical accounts giving various explanations for the bump, and supports the use of a variety of methods of identity construction as possible explanations for the bump phenomenon. It shows that varying locations of the bump could be due to different methods of activating memories or differences in memory types. Future research could examine memory functions and the measure of the self, along with the role of SDMs in the association of identity and the bump. New research could also be conducted on the salience of identity in memories, and the significance of goals in SDM formation. The role of SDMs in helping familiarize an individual to age-related changes could be investigated.

The authors highlight a gap in the research for which, if any, theoretical account offers the best explanation for the bump. The reviewed studies do not provide enough evidence to construct a clear understanding of the bump and its location and formation using different types of memory assessments. There is a need to conduct further studies investigating methods of memory activation, different types of memories activated, and theories for the bump, particularly to compare the plausibility of several theoretical accounts simultaneously in a single study. A novel research strategy could be developed for use in a large study to determine if the narrative/identity account, or cultural life script account, better explains the bump.

### Strengths/Limitations

This review provides a foundation for a more transparent understanding of the relative plausibility of theoretical accounts explaining the bump as reflected in the research. Since the temporal location of the bump varies according to memory activation method, the authors present the bump’s most widely accepted location. This review includes studies in various languages and geographical locations, and with differing population characteristics and lengths. The researchers conducted an extensive quality assessment exercise for study inclusion.

A limitation of this systematic review is simply the heterogeneity of the pool of studies regarding research design, time period conducted, sample size, sample characteristics, intervention strategies implemented during different time periods, and assessment method. Methods used to examine the bump depend on self-report measures, and cannot be evaluated for accuracy of recall due to the potential for self-report bias, which may influence results. The 14 criteria checklist [55] used to assess the quality of studies with diverse designs has inherent limitations. Although it permits a comparison among studies for quality, it gives no guidance for what score is considered “good,” or represents a satisfactory level of internal validity. The authors adopted this checklist based on its use in other published systematic reviews [146, 147].

## Conclusion

This systematic review provides a comprehensive summary of the empirical research on the reminiscence bump published between 1988 and 2017. Findings illustrate that the cuing method and important memories method were widely used to induce memories. Results indicate the overall temporal location of the bump to be between 10–30 years of age for the important memories method, 5–30 years of age for word cuing methods, and 6–39 years of age for studies using life scripts. Both the narrative/identity and cultural life scripts accounts received a fair amount of support in explaining the occurrence of the bump. The authors indicate a need for further research in identifying: (a) the theoretical account offering the most comprehensive explanation for the bump, and (b) the most accurate method(s) of memory activation. The strengths and limitations of both accounts of the bump (i.e., the narrative/identity account and cultural life script account) and suggestions for future studies are discussed. The current, empirical evidence on the bump summarized in this paper could be valuable for researchers and professionals in the fields of cognitive psychology and neuroscience.

## Supporting information

S1 Table Showing Key words and alternative words. Computer-based searches were conducted to search nine databases. In each search, derivatives of “reminiscence bump” were combined using the Boolean OR operator and wildcards.
(DOCX)

S2 Table Databases searched for the systematic review. A search of nine databases gave a total of 523 research articles.
(DOCX)

S3 Table Showing quality assessment of quantitative studies included in this systematic review (n = 68). The detailed quality assessment of all included studies was carried out through a 14 criteria given by Kmet, Lee, and Cook.
(DOCX)

S4 Table PRISMA checklist. A PRISMA checklist showing various section of this review and page numbers on which these sections are reported.
(DOCX)

S1 Fig Geographical distribution of all 68 studies examined in this systematic review. The clustering of studies on the basis of geographical location shows that most of the studies (n = 19) were conducted in USA.
(TIF)

S2 Fig Temporal distribution of all 68 studies examined in this systematic review. The clustering of included studies in three groups; 1988 to 1999; 2000 to 2010, and 2011 to 2017.
(TIF)

S1 File PROSPERO protocol. Review protocol registered in PROSPERO (International prospective register of systematic reviews).
(PDF)
