# Supplementary material for: Understanding the reminiscence bump: A systematic review
Source: PLoS One. 2018 Dec 11;13(12):e0208595. doi: 10.1371/journal.pone.0208595 (PMC6289446; doi:10.1371/journal.pone.0208595)
Supplement: S1 Table — Computer-based searches were conducted to search nine databases. In each search, derivatives of “reminiscence bump” were combined using the Boolean OR operator and wildcards. (DOCX) [file pone.0208595.s001.docx]

**S1 Table. Showing Key words and Alternative Words.**

| **Key word** |  | **Alternative word/s** |
| --- | --- | --- |
| Reminiscence bump | OR | Reminiscence*  Bump  Peak  Surge  Blip  reminiscence effect*  reminiscence component |
