# Supplementary material for: Understanding the reminiscence bump: A systematic review
Source: PLoS One. 2018 Dec 11;13(12):e0208595. doi: 10.1371/journal.pone.0208595 (PMC6289446; doi:10.1371/journal.pone.0208595)
Supplement: S2 Table — A search of nine databases gave a total of 523 research articles. (DOCX) [file pone.0208595.s002.docx]

**S2 Table. Databases Searched for the Systematic Review.**

| **Database** | **Temporal period covered (Start date reflects the year in which each database was established. End date is the date in which the search was limited to)** | **Number of articles obtained** | **Number of articles retained** |
| --- | --- | --- | --- |
| Ovid MEDLINE | 1946 | 40 | 27 |
| Ovid Embase | 1974 | 57 | 36 |
| Ovid Emcare | 1995 | 20 | 13 |
| CINAHL Plus (EBSCOhost) | 1937 | 14 | 10 |
| Proquest Central | 1938 | 4 | 4 |
| PsycInfo | 1806 | 96 | 51 |
| Scopus | 2004 | 88 | 52 |
| Pubmed | 1996 | 50 | 32 |
| ScienceDirect | 1997 | 154 | 7 |
| Total | | 523 | 232 |
