# Supplementary material for: Understanding the reminiscence bump: A systematic review
Source: PLoS One. 2018 Dec 11;13(12):e0208595. doi: 10.1371/journal.pone.0208595 (PMC6289446; doi:10.1371/journal.pone.0208595)
Supplement: S3 Table — The detailed quality assessment of all included studies was carried out through a 14 criteria given by Kmet, Lee, and Cook. (DOCX) [file pone.0208595.s003.docx]

**S3 Table. Showing Quality Assessment of Quantitative Studies Included in this Systematic Review (N=68).**

| Sr. No | Criteria | (Alea, Ali, & Marcano, 2014) | (Dorthe Berntsen & Rubin, 2004) | (Bohn & Berntsen, 2011) | (Chu & Downes, 2000) | (Conway & Haque, 1999) | (Conway, Wang, Hanyu, & Haque, 2005) | (Copeland, Radvansky, & Goodwin, 2009) | (Davison & Feeney, 2008) | (Demiray, Gülgöz, & Bluck, 2009) | (Denver, Lane, & Cherry, 2010) | (Dickson, Pillemer, & Bruehl, 2011) | (Ece & Gülgöz, 2014) | (Elnick, Margrett, Fitzgerald, & Labouvie-Vief, 1999) | (Erdoğan, Baran, Avlar, Taş, & Tekcan, 2008) |
| --- | --- | --- | --- | --- | --- | --- | --- | --- | --- | --- | --- | --- | --- | --- | --- |
| 1 | Question / objective sufficiently described? | 2 | 2 | 1 | 1 | 2 | 2 | 2 | 2 | 2 | 2 | 2 | 2 | 2 | 2 |
| 2 | Study design evident and appropriate? | 2 | 1 | 2 | 1 | 2 | 2 | 2 | 2 | 2 | 2 | 2 | 2 | 2 | 2 |
| 3 | Method of subject/comparison group selection or source of information/input variables described and appropriate? | 2 | 2 | 2 | 2 | 2 | 2 | 1 | 2 | 2 | 2 | 2 | 2 | 2 | 2 |
| 4 | Subject (and comparison group, if applicable) characteristics sufficiently described? | 1 | 2 | 1 | 1 | 2 | 2 | 0 | 1 | 1 | 2 | 2 | 2 | 2 | 2 |
| 5 | If interventional and random allocation was possible, was it described? | 0 | 0 | 0 | 0 | 0 | 0 | 0 | 0 | 0 | 0 | 0 | 0 | 0 | 0 |
| 6 | If interventional and blinding of investigators was possible, was it reported? | N/A | N/A | N/A | N/A | N/A | N/A | N/A | N/A | N/A | N/A | N/A | N/A | N/A | N/A |
| 7 | If interventional and blinding of subjects was possible, was it reported? | N/A | N/A | N/A | N/A | N/A | N/A | N/A | N/A | N/A | N/A | N/A | N/A | N/A | N/A |
| 8 | Outcome and (if applicable) exposure measure(s) well defined and robust to measurement / misclassification bias? Means of assessment reported? | 2 | 2 | 1 | 2 | 2 | 2 | 2 | 2 | 2 | 2 | 2 | 1 | 2 | 2 |
| 9 | Sample size appropriate? | 2 | 2 | 2 | 2 | 2 | 2 | 2 | 2 | 2 | 2 | 2 | 2 | 2 | 2 |
| 10 | Analytic methods described/justified and appropriate? | 2 | 2 | 1 | 2 | 2 | 2 | 2 | 2 | 2 | 2 | 2 | 2 | 2 | 2 |
| 11 | Some estimate of variance is reported for the main results? | 1 | 1 | 1 | 2 | 2 | 2 | 2 | 2 | 2 | 1 | 1 | 2 | 1 | 0 |
| 12 | Controlled for confounding? | 1 | 1 | 1 | 1 | 1 | 1 | 1 | 1 | 1 | 1 | 1 | 1 | 1 | 1 |
| 13 | Results reported in sufficient detail? | 2 | 2 | 2 | 2 | 2 | 2 | 2 | 2 | 2 | 2 | 2 | 2 | 2 | 2 |
| 14 | Conclusions supported by the results? | 2 | 2 | 2 | 2 | 2 | 2 | 2 | 2 | 2 | 2 | 2 | 2 | 2 | 2 |
| Total sum | | 19 | 19 | 16 | 18 | 21 | 21 | 18 | 20 | 20 | 20 | 20 | 20 | 20 | 19 |
| Total possible sum | | 24 | 24 | 24 | 24 | 24 | 24 | 24 | 24 | 24 | 24 | 24 | 24 | 24 | 24 |
| Summary score | | 0.79 | 0.79 | 0.67 | 0.75 | 0.88 | 0.88 | 0.75 | 0.83 | 0.83 | 0.83 | 0.83 | 0.83 | 0.83 | 0.79 |

Yes = 2, Partial = 1, No = 0, NA = not applicable.

| Sr. No. | Criteria | (Fitzgerald, 1988) | (Fitzgerald, 1996) | (Fromholt & Larsen, 1991) | (Fromholt et al., 2003) | (Gidron & Alon, 2007) | (Glück & Bluck, 2007) | (Haque & Hasking, 2010) | (Holmes & Conway, 1999) | (Jansari & Parkin, 1996) | (SMJ Janssen, Chessa, & Murre, 2003) | (Steve Janssen, Chessa, & Murre, 2005) | (S. M. Janssen, Chessa, & Murre, 2007) | (S. M. Janssen, Murre, & Meeter, 2008) | (S. M. Janssen & Murre, 2008) | (S. M. Janssen, Gralak, & Murre, 2011) |
| --- | --- | --- | --- | --- | --- | --- | --- | --- | --- | --- | --- | --- | --- | --- | --- | --- |
| 1 | Question / objective sufficiently described? | 1 | 2 | 2 | 2 | 2 | 2 | 2 | 1 | 2 | 2 | 2 | 2 | 2 | 2 | 2 |
| 2 | Study design evident and appropriate? | 1 | 2 | 2 | 2 | 2 | 2 | 2 | 1 | 2 | 1 | 2 | 2 | 2 | 2 | 2 |
| 3 | Method of subject/comparison group selection or source of information/input variables described and appropriate? | 2 | 1 | 2 | 2 | 2 | 2 | 2 | 2 | 2 | 1 | 1 | 2 | 2 | 2 | 2 |
| 4 | Subject (and comparison group, if applicable) characteristics sufficiently described? | 2 | 1 | 2 | 2 | 1 | 1 | 1 | 1 | 1 | 1 | 1 | 1 | 1 | 1 | 1 |
| 5 | If interventional and random allocation was possible, was it described? | 0 | 0 | 0 | 0 | 0 | 0 | 0 | 0 | 0 | 1 | N/A | N/A | N/A | N/A | N/A |
| 6 | If interventional and blinding of investigators was possible, was it reported? | N/A | N/A | N/A | N/A | N/A | N/A | N/A | N/A | N/A | N/A | N/A | N/A | N/A | N/A | N/A |
| 7 | If interventional and blinding of subjects was possible, was it reported? | N/A | N/A | N/A | N/A | N/A | N/A | N/A | N/A | N/A | N/A | N/A | N/A | N/A | N/A | N/A |
| 8 | Outcome and (if applicable) exposure measure(s) well defined and robust to measurement / misclassification bias? Means of assessment reported? | 1 | 2 | 2 | 2 | 2 | 2 | 2 | 2 | 2 | 2 | 2 | 2 | 2 | 2 | 2 |
| 9 | Sample size appropriate? | 2 | 2 | 2 | 2 | 2 | 2 | 2 | 1 | 2 | 2 | 2 | 2 | 2 | 2 | 2 |
| 10 | Analytic methods described/justified and appropriate? | 1 | 2 | 2 | 2 | 2 | 2 | 2 | 2 | 2 | 2 | 2 | 2 | 2 | 2 | 2 |
| 11 | Some estimate of variance is reported for the main results? | 0 | 1 | 1 | 1 | 0 | 1 | 0 | 1 | 2 | 0 | 1 | 2 | 2 | 2 | 1 |
| 12 | Controlled for confounding? | 0 | 0 | 2 | 0 | 0 | 2 | 0 | 0 | 2 | 0 | 0 | 0 | 0 | 0 | 0 |
| 13 | Results reported in sufficient detail? | 1 | 2 | 2 | 2 | 2 | 2 | 2 | 2 | 2 | 2 | 2 | 2 | 2 | 2 | 2 |
| 14 | Conclusions supported by the results? | 2 | 2 | 2 | 2 | 2 | 2 | 2 | 2 | 2 | 2 | 2 | 2 | 2 | 2 | 2 |
| Total sum | | 13 | 17 | 21 | 21 | 17 | 20 | 17 | 15 | 21 | 16 | 17 | 19 | 19 | 19 | 18 |
| Total possible sum | | 24 | 24 | 24 | 24 | 24 | 24 | 24 | 24 | 24 | 24 | 22 | 22 | 22 | 22 | 22 |
| Summary score | | 0.54 | 0.71 | 0.875 | 0.875 | 0.71 | 0.83 | 0.71 | 0.63 | 0.88 | 0.67 | 0.77 | 0.86 | 0.86 | 0.86 | 0.82 |

| Sr. No. | Criteria | (S. M. Janssen, Rubin, & Jacques, 2011) | (S. M. Janssen, Rubin, & Conway, 2012) | (S. M. Janssen, 2015) | (Ju, Choi, Morris, Liao, & Bluck, 2016) | (Kawasaki, Janssen, & Inoue, 2011) | (Koppel & Berntsen, 2014) | (Koppel & Berntsen, 2016a) | (Koppel & Berntsen, 2016b) | (Krumhansl & Zupnick, 2013) | (Leist, Ferring, & Filipp, 2010) | (Platz, Kopiez, Hasselhorn, & Wolf, 2015) | (Clare J Rathbone, Moulin, & Conway, 2008) | (Clare J. Rathbone, O’Connor, & Moulin, 2017) | (David C Rubin & Matthew D Schulkind, 1997) | (David C. Rubin & Matthew D. Schulkind, 1997) |
| --- | --- | --- | --- | --- | --- | --- | --- | --- | --- | --- | --- | --- | --- | --- | --- | --- |
| 1 | Question / objective sufficiently described? | 2 | 2 | 2 | 2 | 2 | 2 | 2 | 2 | 2 | 2 | 2 | 2 | 2 | 2 | 2 |
| 2 | Study design evident and appropriate? | 2 | 2 | 2 | 2 | 2 | 2 | 2 | 2 | 2 | 2 | 2 | 2 | 2 | 2 | 2 |
| 3 | Method of subject/comparison group selection or source of information/input variables described and appropriate? | 2 | 2 | 2 | 2 | 2 | 1 | 2 | 1 | 2 | 2 | 2 | 2 | 1 | 2 | 2 |
| 4 | Subject (and comparison group, if applicable) characteristics sufficiently described? | 2 | 2 | 1 | 2 | 2 | 2 | 2 | 1 | 2 | 2 | 2 | 1 | 1 | 2 | 2 |
| 5 | If interventional and random allocation was possible, was it described? | 1 | N/A | 0 | 0 | N/A | N/A | 2 | 0 | N/A | N/A | 2 | 2 | 1 | 1 | 2 |
| 6 | If interventional and blinding of investigators was possible, was it reported? | N/A | N/A | N/A | N/A | N/A | N/A | N/A | N/A | N/A | N/A | N/A | N/A | N/A | N/A | N/A |
| 7 | If interventional and blinding of subjects was possible, was it reported? | N/A | N/A | N/A | N/A | N/A | N/A | N/A | N/A | N/A | N/A | N/A | N/A | N/A | N/A | N/A |
| 8 | Outcome and (if applicable) exposure measure(s) well defined and robust to measurement / misclassification bias? Means of assessment reported? | 2 | 2 | 2 | 2 | 2 | 2 | 2 | 2 | 2 | 2 | 2 | 2 | 2 | 2 | 2 |
| 9 | Sample size appropriate? | 2 | 2 | 2 | 2 | 2 | 2 | 2 | 2 | 2 | 2 | 2 | 2 | 2 | 2 | 2 |
| 10 | Analytic methods described/justified and appropriate? | 2 | 2 | 2 | 2 | 2 | 2 | 2 | 2 | 2 | 2 | 2 | 2 | 2 | 2 | 2 |
| 11 | Some estimate of variance is reported for the main results? | 1 | 1 | 1 | 2 | 0 | 1 | 1 | 1 | 1 | 1 | 1 | 2 | 1 | 2 | 2 |
| 12 | Controlled for confounding? | 1 | 0 | 0 | 0 | 0 | 0 | 2 | 0 | 0 | 0 | 1 | 2 | 1 | 2 | 2 |
| 13 | Results reported in sufficient detail? | 2 | 2 | 2 | 2 | 2 | 2 | 2 | 2 | 2 | 2 | 2 | 2 | 2 | 2 | 2 |
| 14 | Conclusions supported by the results? | 2 | 2 | 2 | 2 | 2 | 2 | 2 | 2 | 2 | 2 | 2 | 2 | 2 | 2 | 2 |
| Total sum | | 21 | 19 | 18 | 20 | 18 | 18 | 23 | 17 | 19 | 19 | 22 | 23 | 19 | 23 | 24 |
| Total possible sum | | 24 | 22 | 24 | 24 | 22 | 22 | 24 | 24 | 22 | 22 | 24 | 24 | 24 | 24 | 24 |
| Summary score | | 0.88 | 0.86 | 0.75 | 0.83 | 0.82 | 0.82 | 0.96 | 0.71 | 0.86 | 0.86 | 0.92 | 0.96 | 0.79 | 0.96 | 1 |

| Sr. No. | Criteria | (David C. Rubin, Rahhal, & Poon, 1998) | (David C Rubin & Berntsen, 2003) | (Rybash & Monaghan, 1999) | (Schrauf & Rubin, 1998) | (Schrauf & Rubin, 2001) | (Schubert, 2016) | (Schuman & Corning, 2014) | (Steiner, Pillemer, Thomsen, & Minigan, 2014) | (Svob & Brown, 2012) | (Tekcan, Kaya-Kızılöz, & Odaman, 2012) | (Thomsen & Berntsen, 2008) | (Thomsen, Pillemer, & Ivcevic, 2011) | (Webster & Gould, 2007) | (Wolf & Zimprich, 2016) | (Youichi Maki & Naka, 2006) |
| --- | --- | --- | --- | --- | --- | --- | --- | --- | --- | --- | --- | --- | --- | --- | --- | --- |
| 1 | Question / objective sufficiently described? | 2 | 2 | 2 | 2 | 2 | 2 | 2 | 2 | 2 | 2 | 2 | 2 | 2 | 2 | 2 |
| 2 | Study design evident and appropriate? | 2 | 2 | 2 | 2 | 2 | 2 | 1 | 2 | 2 | 2 | 2 | 2 | 2 | 2 | 2 |
| 3 | Method of subject/comparison group selection or source of information/input variables described and appropriate? | 1 | 2 | 2 | 1 | 1 | 2 | 1 | 2 | 2 | 2 | 2 | 2 | 2 | 2 | 1 |
| 4 | Subject (and comparison group, if applicable) characteristics sufficiently described? | 2 | 2 | 1 | 2 | 1 | 1 | 1 | 1 | 1 | 1 | 1 | 2 | 2 | 2 | 2 |
| 5 | If interventional and random allocation was possible, was it described? | 0 | 1 | 1 | 2 | N/A | 0 | 1 | N/A | 0 | 0 | N/A | N/A | 1 | 1 | 0 |
| 6 | If interventional and blinding of investigators was possible, was it reported? | N/A | N/A | N/A | N/A | N/A | N/A | N/A | N/A | N/A | N/A | N/A | N/A | N/A | N/A | N/A |
| 7 | If interventional and blinding of subjects was possible, was it reported? | N/A | N/A | N/A | N/A | N/A | N/A | N/A | N/A | N/A | N/A | N/A | N/A | N/A | N/A | N/A |
| 8 | Outcome and (if applicable) exposure measure(s) well defined and robust to measurement / misclassification bias? Means of assessment reported? | 2 | 2 | 2 | 2 | 2 | 2 | 2 | 2 | 2 | 2 | 2 | 2 | 2 | 2 | 2 |
| 9 | Sample size appropriate? | 2 | 2 | 2 | 2 | 2 | 2 | 2 | 2 | 2 | 2 | 2 | 2 | 2 | 2 | 2 |
| 10 | Analytic methods described/justified and appropriate? | 1 | 2 | 2 | 2 | 2 | 2 | 1 | 2 | 2 | 2 | 2 | 2 | 2 | 2 | 2 |
| 11 | Some estimate of variance is reported for the main results? | 1 | 2 | 0 | 2 | 2 | 1 | 0 | 1 | 1 | 2 | 1 | 1 | 1 | 2 | 1 |
| 12 | Controlled for confounding? | 0 | 0 | 0 | 0 | 0 | 0 | 0 | 0 | 0 | 0 | 0 | 0 | 0 | 2 | 2 |
| 13 | Results reported in sufficient detail? | 2 | 2 | 2 | 2 | 2 | 2 | 2 | 2 | 2 | 2 | 2 | 2 | 2 | 2 | 2 |
| 14 | Conclusions supported by the results? | 2 | 2 | 2 | 2 | 2 | 2 | 2 | 2 | 2 | 2 | 2 | 2 | 2 | 2 | 2 |
| Total sum | | 17 | 21 | 18 | 21 | 18 | 18 | 15 | 18 | 18 | 19 | 18 | 19 | 20 | 23 | 20 |
| Total possible sum | | 24 | 24 | 24 | 24 | 22 | 24 | 24 | 22 | 24 | 24 | 22 | 22 | 24 | 24 | 24 |
| Summary score | | 0.71 | 0.88 | 0.75 | 0.88 | 0.88 | 0.75 | 0.63 | 0.82 | 0.75 | 0.79 | 0.82 | 0.86 | 0.83 | 0.96 | 0.83 |

| Sr. No. | Criteria | (D. Berntsen, Rubin, & Siegler, 2011) | (Raffard et al., 2010) | (Schlagman, Kliegel, Schulz, & Kvavilashvili, 2009) | (Schroots, Dijkum, & Assink, 2004) | (Raffard et al., 2009) | (Cuervo-lombard et al., 2007) | (Cappeliez, 2008) | (Bernsten & Rubin, 2002) | (Yoichi Maki, Janssen, Uemiya, & Naka, 2013) |
| --- | --- | --- | --- | --- | --- | --- | --- | --- | --- | --- |
| 1 | Question / objective sufficiently described? | 2 | 2 | 2 | 1 | 2 | 1 | 2 | 2 | 2 |
| 2 | Study design evident and appropriate? | 2 | 2 | 2 | 2 | 2 | 2 | 2 | 2 | 2 |
| 3 | Method of subject/comparison group selection or source of information/input variables described and appropriate? | 1 | 1 | 1 | 1 | 1 | 1 | 1 | 1 | 2 |
| 4 | Subject (and comparison group, if applicable) characteristics sufficiently described? | 2 | 2 | 0 | 0 | 2 | 0 | 0 | 2 | 1 |
| 5 | If interventional and random allocation was possible, was it described? | 0 | 0 | 0 | 0 | 0 | 0 | 0 | 0 | 0 |
| 6 | If interventional and blinding of investigators was possible, was it reported? | N/A | N/A | N/A | N/A | N/A | N/A | N/A | N/A | N/A |
| 7 | If interventional and blinding of subjects was possible, was it reported? | N/A | N/A | N/A | N/A | N/A | N/A | N/A | N/A | N/A |
| 8 | Outcome and (if applicable) exposure measure(s) well defined and robust to measurement / misclassification bias? Means of assessment reported? | 2 | 2 | 2 | 2 | 2 | 2 | 2 | 2 | 2 |
| 9 | Sample size appropriate? | 2 | 2 | 2 | 2 | 2 | 2 | 2 | 2 | 2 |
| 10 | Analytic methods described/justified and appropriate? | 2 | 2 | 2 | 2 | 2 | 2 | 2 | 2 | 2 |
| 11 | Some estimate of variance is reported for the main results? | 1 | 2 | 2 | 2 | 2 | 2 | 1 | 1 | 1 |
| 12 | Controlled for confounding? | 0 | 0 | 0 | 0 | 0 | 0 | 0 | 0 | 0 |
| 13 | Results reported in sufficient detail? | 2 | 2 | 2 | 2 | 2 | 2 | 2 | 2 | 2 |
| 14 | Conclusions supported by the results? | 2 | 2 | 2 | 2 | 2 | 2 | 2 | 2 | 2 |
| Total sum | | 17 | 18 | 19 | 15 | 16 | 19 | 16 | 16 | 18 |
| Total possible sum | | 24 | 24 | 24 | 24 | 24 | 24 | 24 | 24 | 24 |
| Summary score | | 0.71 | 0.75 | 0.79 | 0.63 | 0.67 | 0.79 | 0.67 | 0.67 | 0.75 |
