# Supplementary figures and images for: Understanding the reminiscence bump: A systematic review
Source: PLoS One. 2018 Dec 11;13(12):e0208595. doi: 10.1371/journal.pone.0208595 (PMC6289446; doi:10.1371/journal.pone.0208595)

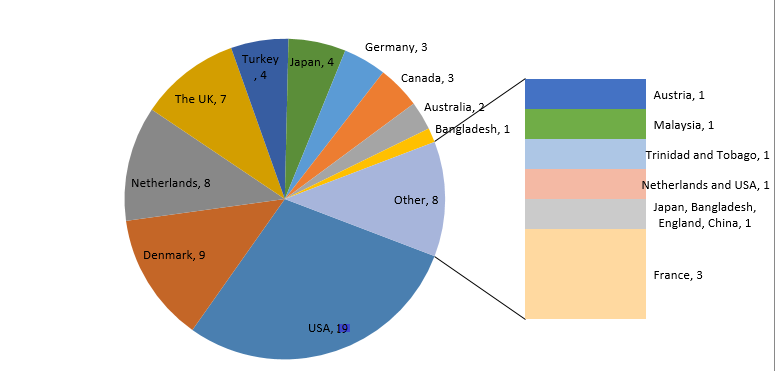

Supplement: S1 Fig — The clustering of studies on the basis of geographical location shows that most of the studies (n = 19) were conducted in USA. (TIF) [file pone.0208595.s005.tif]

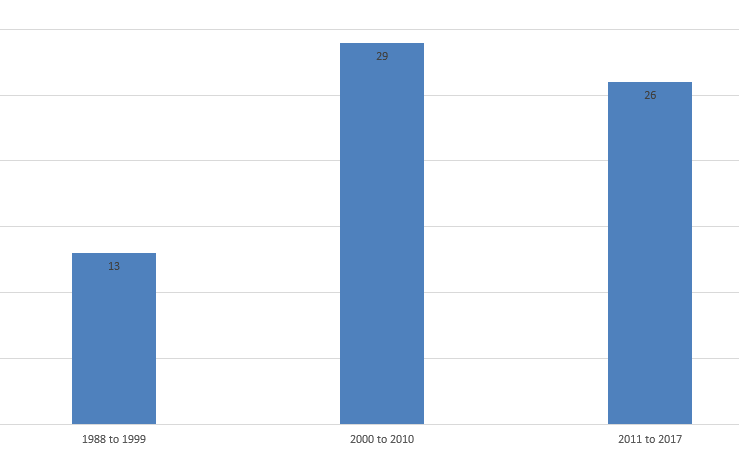

Supplement: S2 Fig — The clustering of included studies in three groups; 1988 to 1999; 2000 to 2010, and 2011 to 2017. (TIF) [file pone.0208595.s006.tif]
