# Supplementary material for: Understanding the reminiscence bump: A systematic review
Source: PLoS One. 2018 Dec 11;13(12):e0208595. doi: 10.1371/journal.pone.0208595 (PMC6289446; doi:10.1371/journal.pone.0208595)
Supplement: S1 File — Review protocol registered in PROSPERO (International prospective register of systematic reviews). (PDF) [file pone.0208595.s007.pdf]

NHS
National Institute for
Health Research

# Understanding the reminiscence bump: A systematic review

*Khadeeja Munawar, Shamsul Haque*

## Citation

Khadeeja Munawar, Shamsul Haque. Understanding the reminiscence bump: A systematic review. PROSPERO 2017 CRD42017076695 Available from:
http://www.crd.york.ac.uk/PROSPERO/display_record.php?ID=CRD42017076695

## Review question

Which theoretical account of reminiscence support has received the maximum support?
In what proportion the accounts are held accountable for the bump?
What are the differences in temporal location of the bump through different methods of memory activation?

## Searches

A systematic search of these databases will be carried out: Ovid MEDLINE, Ovid Embase, Ovid Emcare, EBSCOhost (CINAHL Plus), Proquest Central, PsychInfo, Scopus, Pubmed, and Science direct. Only those search terms which are related to the term “reminiscence bump” will be included.
Keywords will be combined with Boolean and/or operators and wildcards and each database will be searched between dates of inception of each database till 4 Sept, 2017

## Types of study to be included

This review will include following studies: (a) experimental, non-experimental, observational, qualitative studies, and mixed-method studies (b) reviews, (c) articles published in any language, (d) studies conducted on population of any age range, and (e) published in peer-reviewed journals.

## Condition or domain being studied

The domain being studied in this review is "Reminiscence bump" which is an important component of autobiographical memories.
**Autobiographical memories** are memories related to personal live events of individuals (Robinson, 1986) and are clear memories of significant, emotional events and ordinary events (Brewer, 1986; Conway, 1987).

**Reminiscence bump** is a phenomenon when adults recall personal memories from time period when they were 15 to 30 years old as compared to other time periods (Rubin, Wetzler, & Nebes, 1986; Rubin, Rahhal, & Poon, 1998).

## Participants/population

**Inclusion:** The present review will include reminiscence bump related research studies based on various research designs (i.e. experimental, quasi-experimental, qualitative studies, and mixed-method studies).
The participants or populations being studied by this review will belong to all age ranges (no restriction will be applied on the basis of age of participants) as this review will analyse the theoretical account which has received maximum support, the proportion in which every account is held accountable for the bump and the differences in temporal location of the bump through different methods of memory activation.
**Exclusion:** Those studies will be excluded from this review which have not mentioned reminiscence bump (or alternative word/s) in titles or abstracts.

## Intervention(s), exposure(s)

This review will compare the theoretical accounts of reminiscence bump (i.e. cultural life script account, identity formation/ narrative account, cognitive account, cognitive abilities account) to see which theoretical account has received the maximum support.
**Cultural life script account** states that as a result of presence of cultural expectations and scripts in life of

an individual, there is a higher recall of events from the second and third decades of his/her life (Berntsen & Rubin, 2002, 2004; Rubin & Berntsen, 2003).
According to the **identity formation/ narrative account**, memories from early adulthood play a crucial role in identity formation; thus, they occupy a central position in the formation of life narratives (Rathbone, Moulin, Conway & Holmes, 2012).
According to **cognitive account**, adolescence and early adulthood is marked by important events. Those events, which have unique characteristics, go through more rehearsal as compared to preceding and succeeding events; therefore, they are better recalled (Rubin et al., 1998).
The **cognitive abilities account** establishes that it is the ability of humans to better equip themselves to learn, process information, and retain information as they become adolescents and early adults due to maturation of brain which causes maximal cognitive and neurological functioning (Janssen & Murre, 2008).
The review will also compare the methods of activating autobiographical memories (i.e. important memories method, cue word method, and questionnaires) to draw an attention towards differences in temporal location of the bump through different methods of memory activation.
**Important memories method** involves asking participants to report their life's most important memories (Haque & Hasking, 2010; Rubin & Berntsen, 2003; Rubin & Schulkind, 1997a; Thomsen & Berntsen, 2008).
**Cue word method** involves participants describing first personal memory that comes to mind in response to cue words (Conway & Haque, 1999; Crovitz & Schiffman, 1974; Galton, 1879; Hyland & Ackerman, 1988; Jansari & Parkin, 1996; Janssen, Chessa, & Murre, 2005; Maki, Janssen, Uemiya, & Naka, 2013; Rubin, 1982; Rubin & Schulkind, 1997b; Rybash & Monaghan, 1999; Schuman & Corning, 2014).

## Comparator(s)/control

This systematic review will compare the theoretical accounts of reminiscence bump and proportion in which the accounts are held accountable for the bump.
Furthermore, this review will also compare different methods of memory activation to analyze the temporal location of the bump through these methods of memory activation.

## Primary outcome(s)

The main outcome of this review are: 1) support received by the theoretical accounts 2) methods of memory activation 3) temporal location of the reminiscence bump.
**1) Method of memory activation:** The three main methods of activating autobiographical memories are: important memories method, cue word method and questionnaires (already defined in field # 20).
**2) Location of the bump:** It refers to the time period from which most of the memories are reported.
**3) Theoretical accounts of the bump:** There four main theoretical accounts of reminiscence bump; cultural life script account, identity formation/ narrative account, cognitive account and cognitive abilities account (already defined in field # 20).
In order to measure the support received by the theoretical accounts and method of memory activation in most of the included studies, narrative synthesis and then thematic analysis will be carried out. For measuring the location of the reminiscence bump, all the existing reminiscence bumps in included studies will be re-analysed and compared to report the reminiscence bump with each method of memory activation. The temporal location will be analysed along with the thematic analysis.

## Secondary outcome(s)

The secondary outcome of this review is the types of memory recalled. The types of memories activated can be autobiographical memory, memories for public events, and events likely to take place in a prototypical life course . The secondary outcome will be measured like primary outcomes of the review (i.e. following narrative synthesis, a thematic analysis will be carried out). These are not part of inclusion criteria initially.

## Data extraction (selection and coding)

Articles obtained from electronic databases will be exposed to reference citation manager (i.e. EndNote) for screening the title and abstracts for relevance. The reference lists of all articles as well as reviews will be hand searched to ensure inclusions of all relevant studies. This will be done by both reviewers independently and disagreements will be settled through mutual consensus. Duplicates will be removed and studies meeting inclusion criteria will be retained.

In order to extract data, a data extraction chart will be used and data from all included studies will be entered in this chart. This chart will help in assessing methodological quality of included studies and evidence synthesis. Data extraction will be performed by first author and second author will check the data extracted. Extracted information will include: author/year/country, study objective, sample size (n), research design /outcome measures/ method/study type, and findings. Any disagreements between two reviewers will be settled through mutual consensus.

## Risk of bias (quality) assessment

In order to minimise risk of bias, two reviewers will be involved in the review. Both reviewers will work independently for screening relevant studies, extracting data and data synthesis. The discrepancies arising at each and every stage of review will be settled through mutual consensus. The assessment of methodological quality of quantitative studies will be carried out through standardized checklist (Kmet, Lee, & Cook, 2004). The two reviewers will rate quality of individual studies independently of one another. In case of any disagreements in this step, both reviewers will discuss and reach at a unanimous conclusion.

## Strategy for data synthesis

Narrative synthesis will be carried out in order to present the data extracted from included studies in precise manner. This will enable both researchers in using all available data from the narrow pool of research. All available data will be utilized through data extraction, making data extraction chart, and finally summarizing data into qualitative summaries. The stage of familiarizing with the data will be achieved by systematically reviewing each included article multiple times. Initial codes will be formulated through mutual agreement of both reviewers. These codes will assist in generation of themes. Themes will be refined further before giving descriptive titles to them. These themes will be used in final stages of report writing.

## Analysis of subgroups or subsets

None planned

## Contact details for further information

Khadeeja Munawar
khadeeja.munawar14@gmail.com

## Organisational affiliation of the review

Department of Psychology, Jeffrey Cheah School of Medicine and Health Sciences, Monash University Malaysia
http://www.med.monash.edu.my/staff/academic#psychology

## Review team members and their organisational affiliations

Mrs Khadeeja Munawar. Department of psychology, Jeffrey Cheah School of Medicine and Health Sciences, Monash University Malaysia
Assistant/Associate Professor Shamsul Haque. Department of Psychology, Jeffrey Cheah School of Medicine and Health Sciences, Monash University Malaysia

## Anticipated or actual start date

04 September 2017

## Anticipated completion date

21 February 2018

## Funding sources/sponsors

No funding sources are involved in any part of this review.

## Conflicts of interest

None known

## Language

English

## Country
Malaysia

## Stage of review
Review_Ongoing

## Subject index terms status
Subject indexing assigned by CRD

## Subject index terms
Humans; Memory; Mental Recall

## Date of registration in PROSPERO
22 December 2017

## Date of publication of this version
13 February 2018

## Details of any existing review of the same topic by the same authors

## Stage of review at time of this submission

| Stage | Started | Completed |
|---|---|---|
| Preliminary searches | Yes | Yes |
| Piloting of the study selection process | Yes | Yes |
| Formal screening of search results against eligibility criteria | Yes | No |
| Data extraction | Yes | No |
| Risk of bias (quality) assessment | No | No |
| Data analysis | No | No |

## Versions
22 December 2017
13 February 2018

PROSPERO
This information has been provided by the named contact for this review. CRD has accepted this information in good faith and registered the review in PROSPERO. CRD bears no responsibility or liability for the content of this registration record, any associated files or external websites.
